# Aptamers targeting immune checkpoints for tumor immunotherapy: a comprehensive review

**DOI:** 10.3389/fonc.2026.1762902

**Published:** 2026-03-13

**Authors:** Manas Kumar Gedla, Renuka Sai Priya Kotla, Revathy Kumar, Venkatesh Kamesh, Joshua S, Dhanabalan Kamalakannan, Jubilee Ramasamy

**Affiliations:** 1 Department of Pharmacology, PSG College of Pharmacy, Coimbatore, Tamil Nadu, India; 2 Department of Pharmacy Practice, Hindu College of Pharmacy, Guntur, India; 3 Department of Pharmacology, Saveetha College of Pharmacy, Saveetha Institute of Medical and Technical Sciences (SIMATS), Saveetha University, Thandalam, Chennai, India; 4 Department of Pharmaceutical Analysis, Excel College of Pharmacy, Namakkal, Tamil Nadu, India

**Keywords:** aptamers, CTLA-4, immune checkpoints, immunotherapy, nanoparticle delivery, PD-1/PD-L1, resistance mechanisms, T-cell immunoreceptor with Ig and ITIM domains (TIGIT)

## Abstract

Tumor immunotherapy has transformed the cancer treatment paradigm by leveraging the host immune system to identify and eradicate tumor cells in the body. Immune checkpoint inhibitors (ICIs) targeting programmed cell death protein 1 (PD-1), programmed death ligand 1 (PD-L1), and cytotoxic T-lymphocyte-associated antigen 4 (CTLA-4) have achieved significant clinical success. However, major limitations, such as therapeutic resistance, immune-mediated toxicities, and high treatment costs, necessitate the development of alternative and more efficient strategies. Aptamers, short-chain single-stranded nucleic acid ligands with high binding specificity and affinity, have emerged as compelling candidates for cancer therapy due to their superior tissue penetration, reduced immunogenicity, and ease of chemical modification compared to antibody therapies. This review provides an inclusive overview of aptamer-based approaches for targeting immune checkpoints, with a specific emphasis on PD-1/PD-L1 and CTLA-4. In addition, we highlight recent advancements in the engineering of bispecific and multifunctional aptamers, their role in overcoming immune resistance, and their potential to improve therapeutic performance. We also discuss innovative approaches to enhance aptamer stability, bioavailability, and tumor-specific delivery through chemical tailoring and nanoparticle conjugation. Although most aptamer-based checkpoint inhibitors remain in preclinical stages, early phase clinical investigations (primarily with C-X-C motif chemokine ligand 12 (CXCL12)-targeting Spiegelmer NOX-A12 in combination settings, as well as earlier programs such as AS1411 targeting nucleolin) have demonstrated effective inhibition of immune checkpoint signaling, reactivation of T-cell function, and synergistic effects when combined with existing immunotherapies. Preclinical and early phase clinical investigations have demonstrated that aptamers can effectively inhibit immune checkpoint signaling, reactivate T-cell function, and potentiate synergistic effects when combined with existing immunotherapies. By critically evaluating current progress and identifying key translational challenges, this review provides strategic insights into the future development of aptamer-based immunotherapeutic platforms, ultimately guiding the advancement of more precise, cost-effective, and personalized cancer treatment modalities.

## 1 Introduction

**Figure 1:**
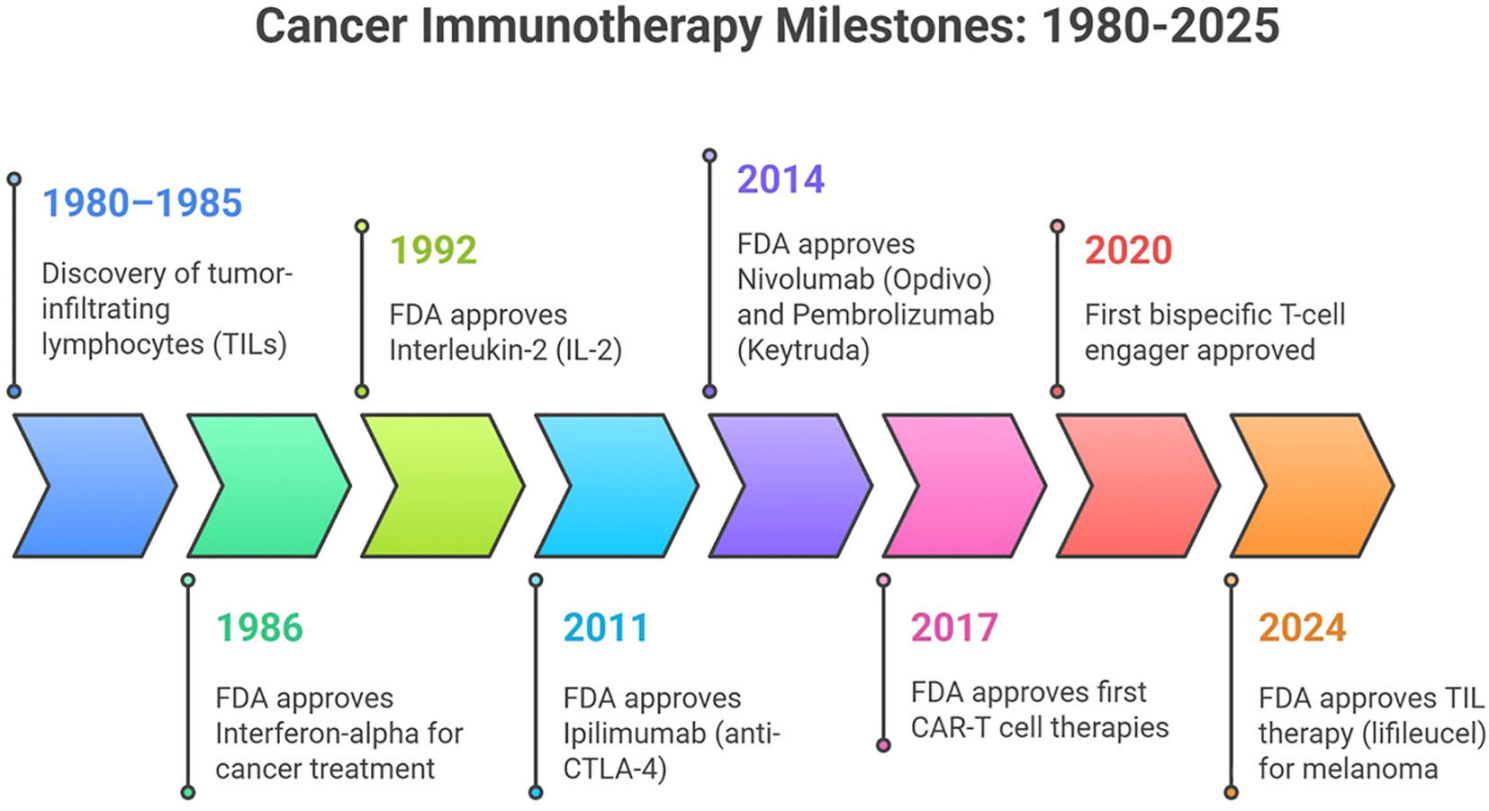
Cancer immunotherapy millstone.

Cancer remains one of the leading causes of mortality worldwide, with over 20 million new cases and 10 million deaths reported in 2022 alone. Projections by the World Health Organization indicate an increase to 28 million new cases annually by 2040 (1). The emergence of cancer immunotherapy in the 1980s marked a major shift in oncology, although the concept of disease-specific immunity dates back to Thucydides’ observations in the 5th century BC (**Figure 1**). Modern immunotherapy gained prominence following the identification of CTLA-4 in 1987 by Brunet et al. and the subsequent approval of the U.S. Food and Drug Administration (FDA) approved interferon-α2-based therapeutics (2). A significant challenge in immunotherapy is treatment resistance, which may be intrinsic or acquired and is often driven by the downregulation of antigen presentation, overexpression of immune checkpoint molecules, or establishment of an immunosuppressive tumor microenvironment (TME). Tumors are generally categorized as “hot” (immune-infiltrated and responsive) or “cold” (poorly infiltrated and unresponsive) (1). Given the heterogeneity of tumor microenvironments and genetic variability, current immunotherapies often yield inconsistent outcomes, underscoring the need for enhanced patient stratification techniques and innovative therapeutic strategies to overcome immune evasion and therapy resistance (3). Aptamers, short DNA or RNA oligonucleotides with high binding specificity, have emerged as promising agents owing to their low immunogenicity, high molecular precision, and lower production costs compared to conventional treatments. Recent studies have demonstrated that aptamer integration into immunotherapy enhances therapeutic outcomes by targeting the critical mechanisms involved in cancer progression and immune evasion (4, 5). Unlike chemotherapy and radiotherapy, which often damage normal cells, immunotherapy aims to activate the immune system to detect and eliminate malignant cells via mechanisms such as immune checkpoint blockade, T cell activation, and cytokine signaling. ICIs targeting PD-1, PD-L1, and CTLA-4 reactivate cytotoxic T cells, improving survival in cancers such as melanoma, non-small cell lung carcinoma, and renal cell carcinoma, with ipilimumab being the first FDA-approved checkpoint inhibitor in 2011 (6–8). However, resistance, immune-related adverse events, and high treatment costs necessitate the development of alternative therapeutic modalities, including aptamer-based approaches (9). Research on aptamer-based immunotherapy has predominantly focused on PD-1/PD-L1, followed by CTLA-4, whereas alternative targets such as NKG2A, NKG2D, and nucleolin are gaining attention. There is an increasing emphasis on bispecific and multifunctional aptamers that target multiple checkpoint pathways. Aptamers, often termed “chemical antibodies,” offer advantages over monoclonal antibodies, including smaller size, tunable structure, reduced immunogenicity, and enhanced tissue penetration. Chemical modification strategies, including PEGylation, further improve the stability and bioavailability of aptamers. The clinical success of pegaptanib (Macugen), an FDA-approved aptamer for age-related macular degeneration, underscores its translational potential. In oncology, aptamers such as AS1411 (targeting nucleolin) have shown promise in breast and lung cancers, whereas NOX-A12 (olaptesed pegol), targeting C-X-C motif chemokine ligand 12 (CXCL12), has enhanced the efficacy of immune checkpoint inhibitors in early phase trials by modulating the tumor microenvironment (TME). Despite significant advancements, most aptamer-based immune checkpoint inhibitors remain preclinical, with only a few (NOX-A12 in combination settings) having entered early phase clinical evaluation. This review aims to provide a thorough examination of the latest progress in aptamer-based immunotherapy, focusing particularly on targeting immune checkpoints, such as PD-1, PD-L1, and CTLA-4. It also explores the design of bispecific and multifunctional aptamers, as well as methods to improve their bioavailability, stability, and tumor penetration through chemical modifications and the use of nanoparticles or immunostimulating agents. By systematically evaluating the current evidence and identifying major challenges, this study offers valuable insights to advance the development of effective and personalized cancer therapies using aptamers.

## 2 Tumor immunotherapy resistance and treatment failure

Tumor immunotherapy has profoundly transformed the landscape of oncology by harnessing the immune system’s ability to recognize and eliminate malignant cells (10, 11). This therapeutic paradigm encompasses immune checkpoint inhibitors directed against PD-1, PD-L1, and CTLA-4, adoptive cell therapies such as CAR-T cell therapy, and various cytokine-based therapies. Collectively, these strategies have achieved unprecedented clinical success, with response rates approaching 50 percent in carefully selected patients with melanoma and non-small cell lung cancer (12, 13). Nevertheless, approximately 60–70 percent of patients with solid tumors ultimately experience disease progression due to the emergence of primary and acquired resistance mechanisms that compromise durable clinical benefits. Primary resistance, defined as the absence of an initial therapeutic response, is often driven by tumor-intrinsic features that impair immune recognition and cytotoxic T cell activation (14, 15). Tumors with a low mutational burden, typically fewer than ten mutations per megabase, generate insufficient neoantigens to elicit a robust antitumor immune response (16). Mutations in the components of the antigen-processing machinery, including B2M and HLA genes, disrupt MHC-I presentation and prevent effective T cell engagement in the TME. Constitutive overexpression of PD-L1 further suppresses T cell activation (17). These features are characteristic of poorly immunogenic malignancies, such as prostate and pancreatic cancers, which exhibit very low response rates to checkpoint blockade therapy (18). Acquired resistance arises after an initial period of clinical benefit and reflects the ability of tumors to adapt to selective immune pressure. Mechanisms include the loss of neoantigens through immunoediting and the upregulation of alternative inhibitory receptors such as TIM-3, LAG-3, VISTA, and TIGIT, which collectively drive T cell exhaustion. Mutations in IFN-γ signaling mediators, including JAK1 and JAK2, blunt PD-L1 induction and impair T cell responsiveness. Dysregulated oncogenic signaling via the MAPK, PI3K–AKT, and WNT–β-catenin pathways enhances immune escape by increasing PD-L1 expression and excluding cytotoxic lymphocytes from the tumor microenvironment. Epigenetic alterations, such as EZH2-mediated histone methylation, suppress chemokine production and reduce MHC-I expression, thereby diminishing immune cell recruitment. Prolonged IFN-γ exposure can paradoxically amplify resistance by inducing the expression of inhibitory ligands, thereby dampening the immune activity (19).

**Figure 2:**
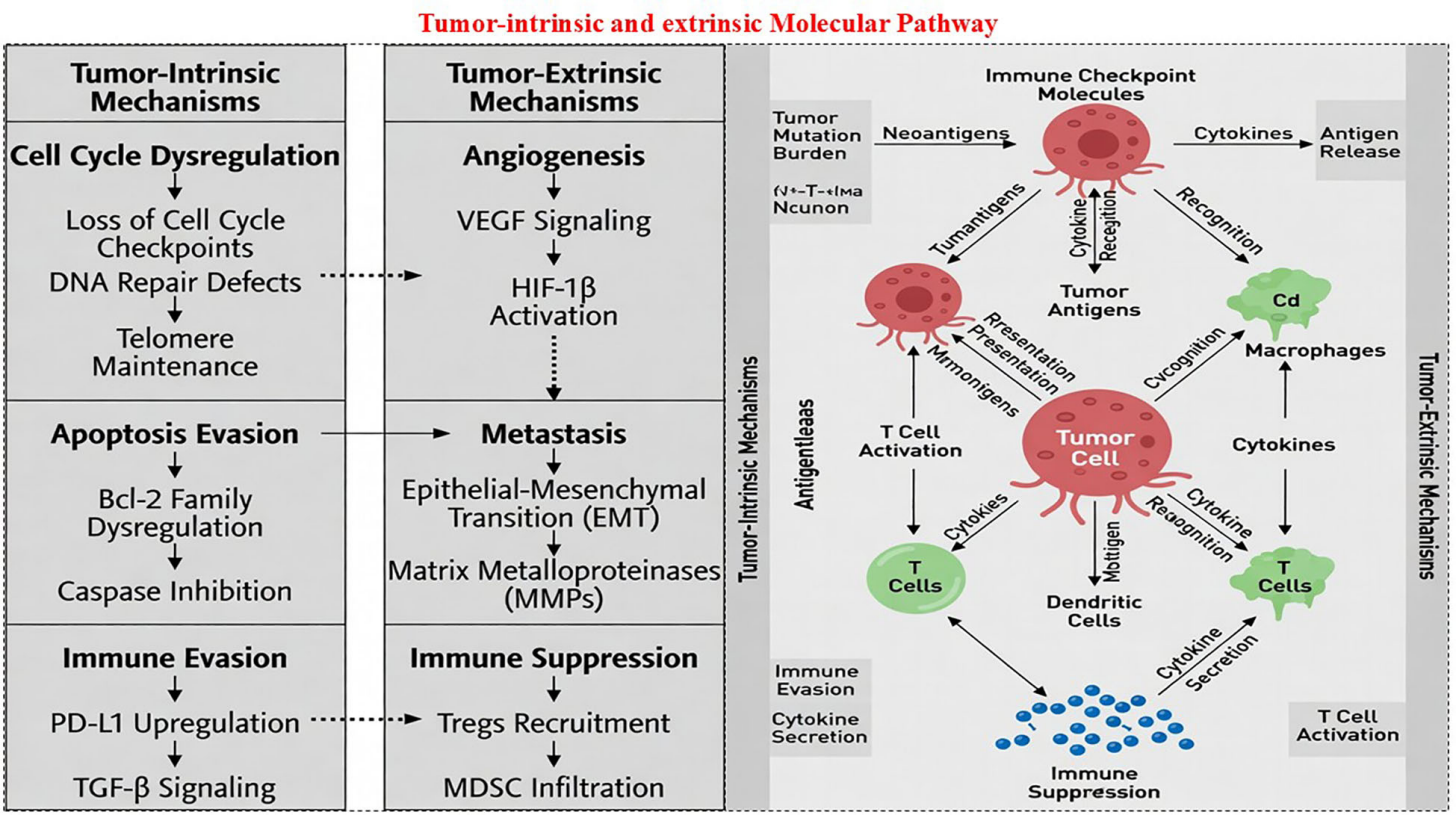
Tumor-intrinsic and tumor-extrinsic molecular pathways.

In addition to tumor-intrinsic mechanisms, tumor-extrinsic factors within the tumor microenvironment exert potent immunosuppressive effects that limit therapeutic efficacy (**Figure 2**). Many tumors display immune desert or “cold” phenotypes, which are characterized by insufficient lymphocyte infiltration and ineffective immune priming. Regulatory T cells secrete IL-10 and TGF-β, MDSCs release ARG1 and PGE_2_, and M2-polarized TAMs inhibit cytotoxic T cell trafficking and function, together creating a profoundly suppressive milieu. CAFs promote dense stromal remodeling, which physically excludes immune cells, whereas VEGF-driven angiogenesis reinforces immunosuppression by inducing inhibitory ligands and checkpoint molecules in endothelial cells. Metabolic dysregulation in the tumor microenvironment further exacerbates this immune dysfunction. IDO-mediated tryptophan depletion and adenosine accumulation under hypoxic conditions impair T cell metabolism and effector functions. Host-related factors further complicate these problems. An unfavorable gut microbiome dominated by Bacteroidales, coupled with reduced levels of beneficial species such as Bifidobacterium and Akkermansia muciniphila, is strongly correlated with poor immunotherapy responses, whereas antibiotic use is associated with decreased survival rates. Age-related immunosenescence and comorbidities, such as obesity, further disrupt the cytokine networks and amplify resistance. Additional mechanisms sustaining immune evasion include epithelial-mesenchymal transition, which promotes T cell exclusion and PD-L1 expression; exosomal PD-L1 secretion, which mediates systemic immunosuppression; and autophagy-driven MHC-I degradation. Compounding these biological barriers are immune-related adverse events that affect most patients who receive combination therapy. These toxicities range from mild fatigue to severe manifestations, such as myocarditis and neutropenia, often necessitating treatment interruption or discontinuation of treatment. Collectively, these multifaceted resistance mechanisms highlight the urgent need for predictive biomarkers, personalized therapeutic strategies, and rationally designed combination regimens. Aptamer-based approaches may specifically address several of these resistance mechanisms through superior tumor penetration (due to small size ~10–30 kDa vs. antibodies ~150 kDa), enabling access to hypoxic or poorly vascularized tumor cores; bispecific designs targeting multiple checkpoints (e.g., PD-1 + LAG-3 or PD-L1 + TIM-3) to counter upregulation of alternative inhibitory pathways; and nanoparticle conjugation for localized, sustained delivery that disrupts metabolic suppression (e.g., adenosine or IDO pathways) or restores antigen presentation via co-delivery of adjuvants or immunostimulatory agents. However, complete antigen loss through immunoediting remains particularly challenging and may require a combination of adoptive cell therapies or neoantigen vaccines. Targeting emerging inhibitory pathways, including TIGIT and TIM-3, alongside microbiome modulation and next-generation drug delivery systems, represents a promising strategy to overcome resistance and achieve more durable clinical responses in tumor immunotherapy (20, 21).

## 3 Tumor microenvironment

**Figure 3:**
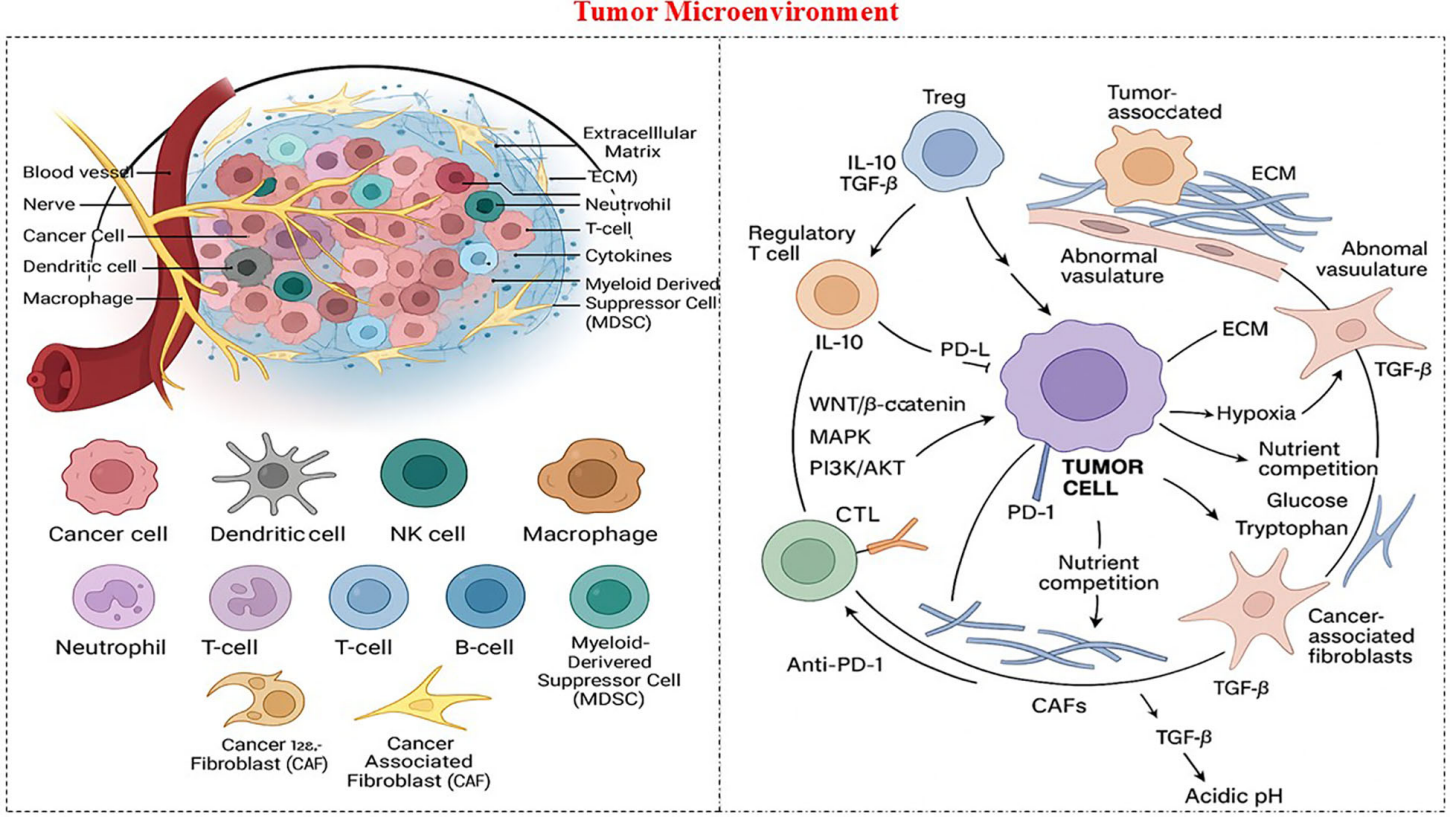
Components of the tumor microenvironment (TME).

The tumor microenvironment is a highly dynamic and heterogeneous ecosystem consisting of malignant cells and a diverse array of non-malignant elements that collectively regulate tumor initiation, progression, metastatic dissemination, and therapeutic resistance, particularly in the context of immunotherapy (**Figure 3**). This complex milieu includes various cellular constituents, such as CD8^+^ cytotoxic T lymphocytes, regulatory T cells, tumor-associated macrophages, myeloid-derived suppressor cells, and tumor-associated neutrophils; stromal components, such as cancer-associated fibroblasts, endothelial cells, and pericytes; and non-cellular elements, including a structurally and biochemically complex extracellular matrix enriched with collagen, fibronectin, and proteoglycans (22, 23). These are further complemented by a repertoire of soluble mediators, notably transforming growth factor-beta, IL-10, IL-6, chemokines such as CXCL12 and CCL2, growth factors including VEGF and FGF, and metabolites such as adenosine and lactate. Together, these components establish an intricate signaling network that orchestrates immune evasion, stromal remodeling, angiogenesis, and metabolic reprogramming, ultimately shaping tumor behavior and influencing therapeutic outcomes (24). Within this regulatory landscape, TAMs often adopt an M2-like pro-tumoral phenotype in response to tumor-derived cytokines, including IL-4 and CSF-1. These cells exert potent immunosuppressive effects through the secretion of IL-10 and TGF-β while simultaneously driving angiogenesis via VEGF and MMP9, remodeling the extracellular matrix through cathepsins and proteases, and facilitating epithelial–mesenchymal transition through TGF-β and TNF-α to enhance metastatic potential (25). MDSCs, recruited by CXCL12 and PGE_2_, contribute to immune suppression by depleting key amino acids such as arginine and tryptophan through the enzymatic activity of ARG1 and IDO, leading to T-cell anergy and apoptosis. TANs skewed toward an N2 pro-tumoral phenotype further intensify this immunosuppressive niche by secreting MMP9 and oncostatin-M to degrade extracellular matrix barriers and stimulate HGF-mediated tumor cell motility. Regulatory T-cells, attracted by CCL22, suppress effector T-cell function via CTLA-4 engagement and IL-2 consumption, thereby diminishing the infiltration and cytotoxic activity of CD8^+^ T-cells within the tumor microenvironment.

The stromal compartment plays a crucial role in reinforcing tumor immune evasion and therapeutic resistance. CAFs, activated from resident fibroblasts or MSCs through TGF-β and PDGF signaling, deposit dense extracellular matrix components that create formidable physical barriers to both immune cell trafficking and drug penetration (26). Additionally, CAFs secrete HGF, SDF-1, and exosomes enriched with miR-21, collectively driving chemoresistance, epithelial–mesenchymal transition, and enhanced DNA repair capacity, all of which contribute to radioresistance. MSCs, recruited in response to tissue damage or irradiation, release fatty acids and cytokines, such as TNF-α, to further recruit immunosuppressive macrophages, expand cancer stem cell populations through CCL5–AR signaling, and differentiate into CAFs that support neovascularization and paracrine survival circuits (27). The noncellular matrix provides structural support and serves as a dynamic regulatory scaffold. Under hypoxic conditions, characteristic of poorly vascularized solid tumors, ECM remodeling is accompanied by the stabilization of HIF-1α, which induces PD-L1 expression and generates nutrient-deprived niches that promote the accumulation of immunosuppressive adenosine through CD39 and CD73 activity (28). These alterations facilitate the transfer of resistance factors, including multidrug transporters, via exosomes and activate adaptive survival pathways that enable tumors to withstand the therapeutic pressure. Taken together, these coordinated cellular, stromal, and molecular interactions converge to establish a profoundly immunosuppressive and treatment-refractory tumor microenvironment. The TME represents a major obstacle to effective immunotherapy by evading immune checkpoints, inducing T cell exhaustion, and rewiring the metabolic and signaling pathways. These elements create significant barriers to aptamer efficacy. The dense extracellular matrix and high interstitial pressure limit diffusion and penetration, particularly in hypoxic cores. Low pH and altered ionic strength can impair aptamer folding and binding affinities. Hypoxia-induced upregulation of alternative checkpoints and immunosuppressive metabolites further complicates the single-agent blockade. Consequently, therapeutic strategies aimed at reprogramming the TME, such as targeting VEGF to normalize aberrant vasculature or inhibiting IDO to restore metabolic homeostasis, hold significant promise for overcoming resistance and enhancing the efficacy of immunotherapeutic approaches in patients with NSCLC (29). Aptamer-nanoparticle conjugates, PEGylation, and TME-targeting aptamers (e.g., against CXCL12, VEGF, or cancer-associated fibroblasts) offer promising strategies for improving penetration, maintaining structural integrity under physiological stress, and reprogramming the immunosuppressive milieu.

## 4 Aptamers

### 4.1 Structural features of DNA and RNA aptamers

**Figure 4:**
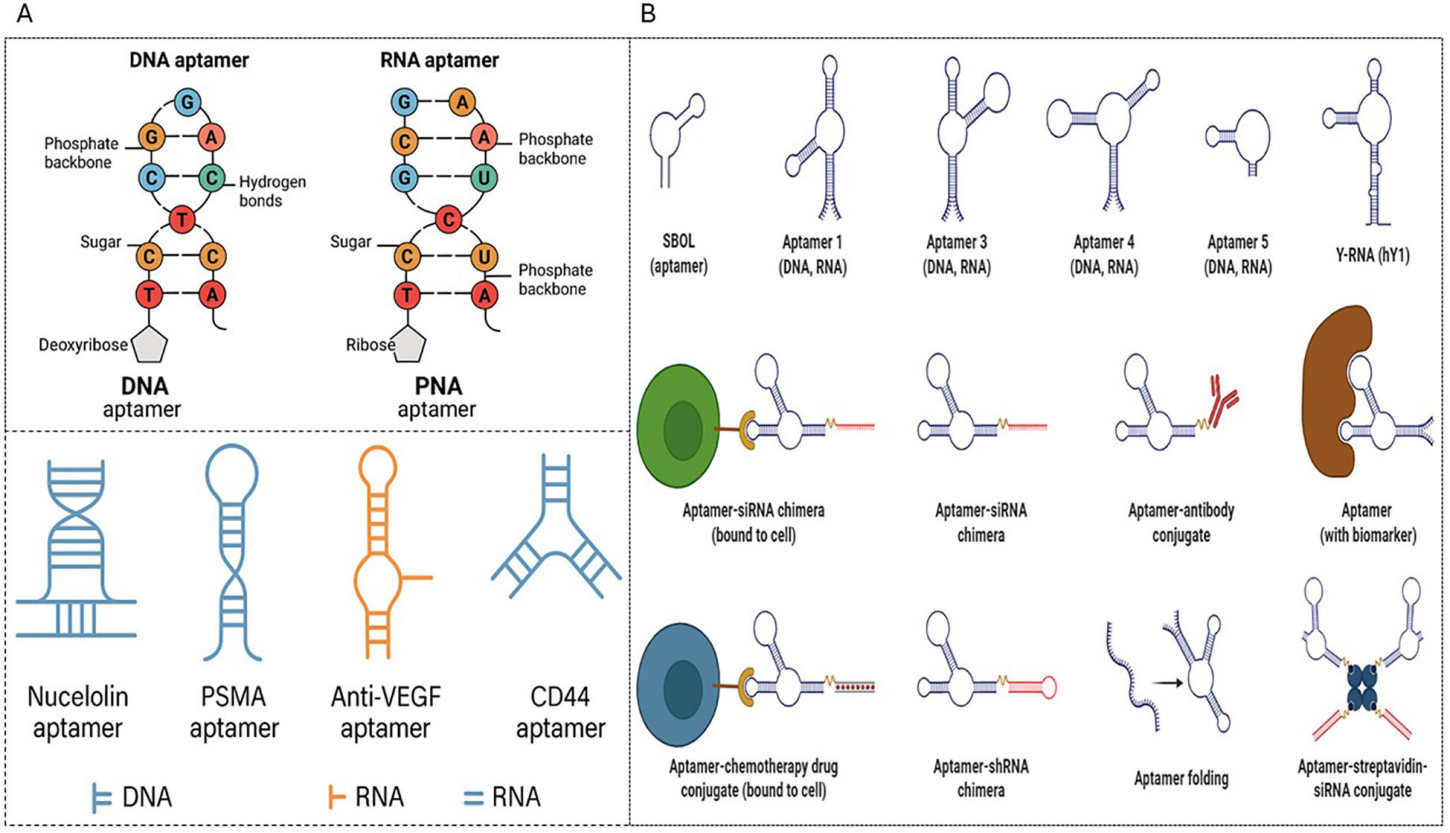
Structural types and functional applications of aptamers. **(A)** Structural comparison of DNA, RNA, and PNA aptamers **(B)** Schematic representation of aptamer secondary structures and engineered formats, including aptamer -siRNA chimeras, antibody and drug conjugates, and other functional constructs for targeted therapy.

Aptamers are short, single-stranded DNA or RNA oligonucleotides, typically 20–100 nucleotides long, that fold into stable three-dimensional structures capable of high-affinity and high-specificity molecular recognition, as illustrated in **Figure 4A**, **Table 1**. Their structural versatility arises from intramolecular base pairing, stacking interactions, and tertiary contacts, which generate diverse structural motifs. Secondary motifs include hairpin stems–loops that act as stable scaffolds, internal bulges that introduce conformational flexibility and create binding pockets, and pseudoknots formed by long-range base pairing that stabilize complex tertiary folds. A particularly important motif is the G-quadruplex, a guanine-rich structure formed by planar G-quartets stacked through Hoogsteen hydrogen bonds and stabilized by monovalent cations such as K^+^, imparting exceptional stability and affinity, as observed in the classic thrombin-binding aptamer (TBA) structure. RNA aptamers exhibit additional structural richness owing to the 2′-hydroxyl group, which favors A-form helices and enables tertiary interactions, such as GNRA tetraloops, A-minor motifs, ribose zippers, and kissing loop elements that stabilize compact globular folds reminiscent of protein-binding pockets. These intricate secondary and tertiary interactions underpin the remarkable ability of aptamers to fold into unique, target-specific architectures that rival or surpass antibodies in terms of selectivity and binding strength (30, 31).

**Table 1:** Structural features of DNA and RNA aptamers.

Feature	Description	Structural role	Functions
General Structure	Short single-stranded DNA or RNA (20–100 nt) that fold into stable 3D conformations	Enable high-affinity and high-specificity binding to molecular targets	Folding driven by base pairing and stacking interactions
Hairpin Stem–Loops	Double-stranded stem with a loop at the end	Provide structural stability and act as scaffolds for tertiary organization.	Common in both DNA and RNA aptamers
Bulges (asymmetric/symmetric)	Unpaired regions within a helical stem	Introduce flexibility; form unique binding pockets	Allow for structural adaptability and target accommodation
Pseudoknots	Formed when loop regions pair with distant complementary sequences	Bring noncontiguous regions together; stabilize tertiary structures	Frequently observed in high-affinity RNA aptamers
G-Quadruplexes	Guanine-rich sequences forming planar G-quartets stacked via Hoogsteen H-bonds, stabilized by cations (e.g., K^+^)	Confer exceptional structural stability; create unique binding surfaces	Common in DNA aptamers (e.g., thrombin-binding aptamer)
RNA-Specific Structural Features	2′-hydroxyl group promotes A-form helices, compact folds, and complex tertiary motifs	Allows formation of intricate pseudoknots and tetraloops	GNRA tetraloops often selected during SELEX

### 4.2 Types and functional classes of aptamers

Aptamers are classified into several distinct types based on their molecular composition and function, as shown in **Figure 4B** and listed in **Table 2**. DNA aptamers typically form hairpins and G-quadruplexes, offering excellent thermal stability and ease of chemical synthesis for diagnostic and therapeutic applications. RNA aptamers exhibit more complex tertiary structures and often achieve higher binding affinities but require chemical modifications for nuclease resistance *in vivo*. Spiegelmers (L-aptamers) are mirror-image nucleic acids composed of L-nucleotides, making them highly resistant to enzymatic degradation and ideal for therapeutic use (32). Peptide–aptamer hybrids combine the molecular recognition of aptamers with peptide functionalities, thereby enhancing the cellular delivery and multivalency of the resulting conjugates. Functionally, aptamers can target small molecules, forming compact binding pockets for metabolites and drugs; proteins and cell surfaces, enabling targeted imaging and therapy; or can be engineered as nanostructured aptamers, assembled into multivalent or branched architectures that increase avidity and allow multifunctional applications. This structural and functional diversity positions aptamers as a versatile platform that bridges molecular recognition, synthetic biology, and translational medicine (33).

**Table 2:** Types and functional classes of aptamers.

Aptamer type	Subtype	Description	Advantages	Mechanisms	Examples
Nucleic Acid	DNA	ssDNA with stable folds like G-quadruplexes	High stability, low cost	Binds via 3D shape complementarity	AS1411 (cancer therapy)
Nucleic Acid	RNA	ssRNA with flexible structures	Broad target range	Conformational changes upon binding	Pegaptanib (AMD treatment)
Peptide	–	Loops in protein scaffolds	High affinity (up to 1000x free peptides)	Entropy reduction for binding	EphA2 inhibitors
Modified	Spiegelmers	L-enantiomer RNA/DNA	Nuclease resistance	Mirror-image configuration	NOX-E36 (chemokine neutralization)
Modified	Circular	Cyclized oligonucleotides	Enhanced durability	Protection from exonucleases	PD-L1/CTLA-4 bispecifics
Cleaved	Hairpin-loop	Split with retained loops	Reduced non-specificity	Ternary complex formation	Small molecule sensors
Cleaved	Three-way linkage	Central binding optimization	Improved sensitivity	Fragment assembly on target	Cocaine detection aptamers
Functional	Bispecific	Dual/multiple sites	Synergistic effects	Bridging targets	CD16/c-Met for NK lysis

### 4.3 Aptamer target recognition and binding mechanisms

**Figure 5:**
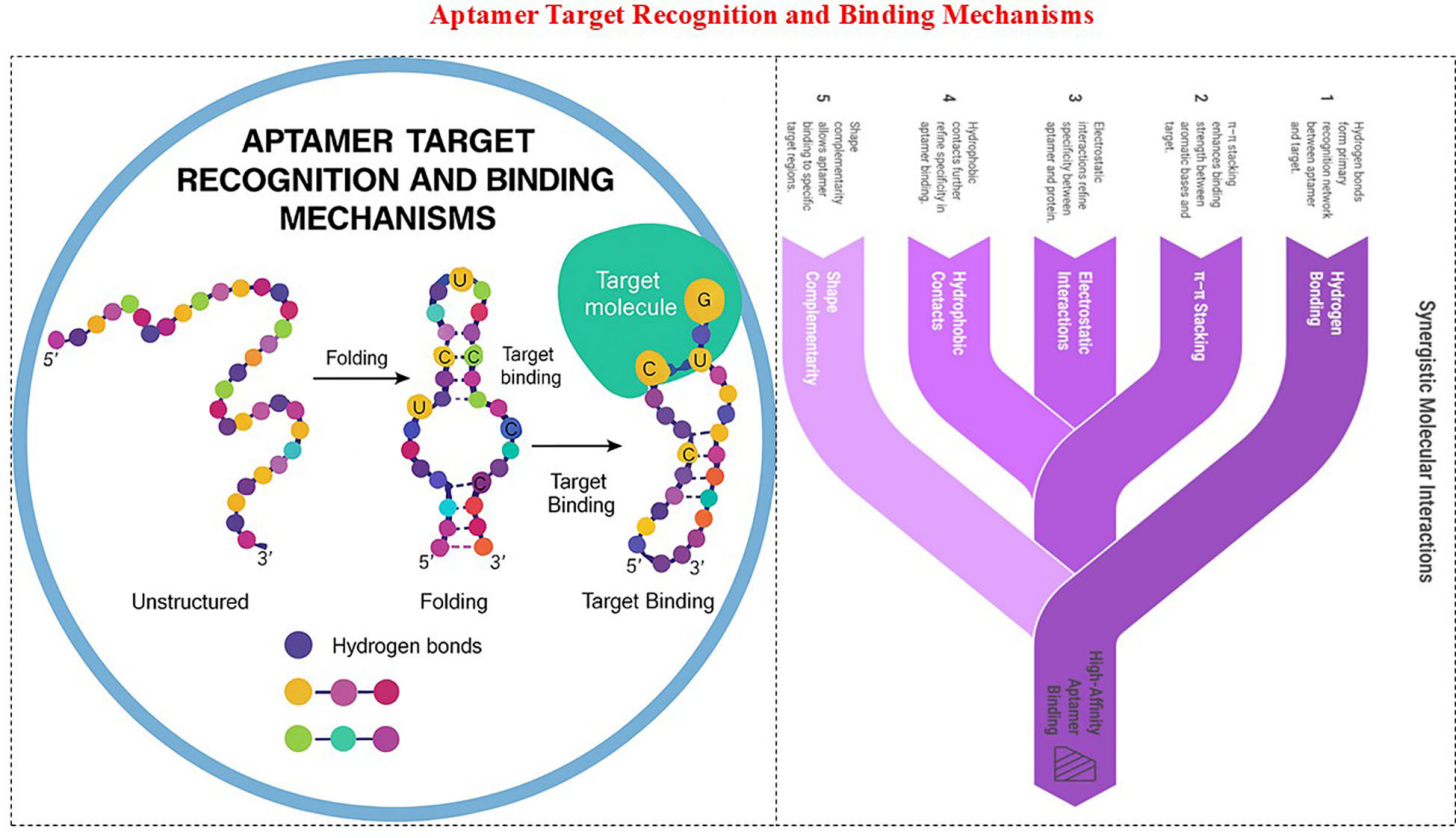
Aptamer target recognition and binding mechanism.

Structural Basis of Recognition Aptamers recognize their targets through precise folding into stable three-dimensional (3D) architectures that generate unique molecular interfaces complementary to their ligands (**Figure 5**). Their single-stranded DNA or RNA sequences self-assemble into structural motifs, such as hairpin stem loops, asymmetric bulges, pseudoknots, GNRA tetraloops, A-minor motifs, and G-quadruplexes, each of which contributes to the overall structural integrity and formation of the binding pocket. These motifs mimic protein active sites or antibody paratopes, allowing aptamers to engage a wide range of targets, including small molecules, peptides, proteins, cells, and nanomaterials with remarkable specificity. For example, G-quadruplexes, stabilized by Hoogsteen hydrogen bonds and monovalent cations (K^+^), provide rigid platforms for high-affinity binding, whereas pseudoknots stabilize complex tertiary folds that can occupy enzyme clefts or surface epitopes of proteins. Recognition may occur through conformational selection, where the aptamer exists in multiple folded states and selects the most compatible conformation upon ligand encounter, or through induced fit, where target binding triggers structural rearrangements to optimize interactions (34).

Molecular Interactions and Binding Mechanisms Once the aptamer folds into its functional architecture, molecular interactions drive and stabilize the complex formation. Hydrogen bonding between nucleobases and target functional groups forms the primary recognition network, whereas π–π stacking between aromatic bases and planar regions of the target enhances the binding strength. Electrostatic interactions between the negatively charged phosphate backbone and positively charged residues on protein surfaces, as well as hydrophobic contacts and shape complementarity, further refine the specificity. In small-molecule aptamers, stacking and hydrogen bonding define the tight encapsulation of ligands. In protein-targeting aptamers, shape complementarity allows binding to active sites, allosteric regions, or conformational epitopes, often blocking the signaling pathways or inducing receptor internalization. Cell-SELEX-derived aptamers typically bind to cell surface proteins, glycans, and conformational epitopes without prior target identification. These synergistic interactions enable aptamers to achieve nanomolar to picomolar affinities comparable to or exceeding those of monoclonal antibodies while retaining reversibility, chemical stability, and tunability for diagnostic, therapeutic, and nanobiotechnological applications (35).

### 4.4 Principles of aptamer screening (SELEX)

**Figure 6:**
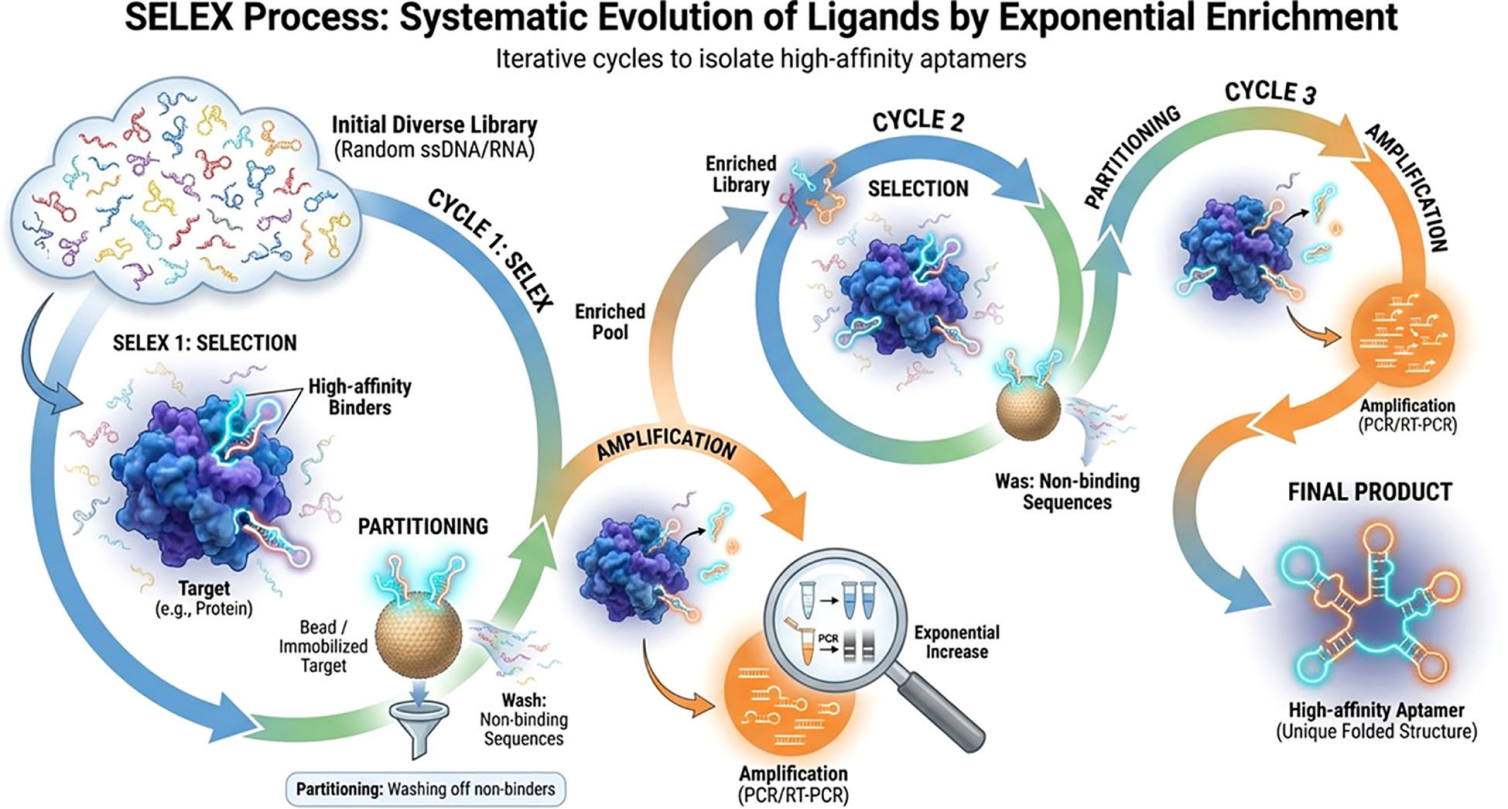
Principles of aptamer screening using the SELEX process.

Aptamers are single-stranded DNA or RNA molecules that can fold into unique three-dimensional conformations, conferring high specificity and affinity for diverse targets, including proteins, small molecules and cells. The conventional method for aptamer discovery is the Systematic Evolution of Ligands by Exponential Enrichment (SELEX), an iterative *in vitro* selection process (**Figure 6**). SELEX begins with a highly diverse library containing approximately 10^12^–10^14^ random oligonucleotide sequences. This library is incubated with the target molecule, and sequences that successfully bind are separated from non-binding sequences and subsequently amplified by polymerase chain reaction (PCR) to generate an enriched pool for the next round of selection. Multiple cycles of binding, partitioning, and amplification, typically between five and 15, are performed to progressively enrich sequences with the highest affinity and specificity (36). Although SELEX remains the gold standard for aptamer discovery, it is often time-consuming, labor-intensive, and limited by experimental throughput, with each round taking several days to weeks, and only a small fraction of the candidate sequences is ultimately validated (37). To address these limitations, bioinformatics approaches have been integrated into SELEX pipelines. Computational tools can predict aptamer secondary and tertiary structures, estimate binding affinities, and rank candidates before or during experimental rounds. High-throughput SELEX, combined with next-generation sequencing and computational motif analysis, enables the rapid identification of enriched sequences and conserved binding motifs across multiple selection rounds. Structure prediction platforms, such as RNAfold, Mfold, and RNAComposer, facilitate the pre-screening of structural candidates, whereas molecular docking and machine-learning algorithms allow the in silico ranking of potential binders. These bioinformatics-driven enhancements streamline the SELEX workflow by pre-filtering libraries, optimizing pool design, and characterizing enriched sequences *post hoc*, thereby reducing the experimental workload and accelerating aptamer discovery (38).

### 4.5 Computational strategies for *in-silico* aptamer selection

**Figure 7:**
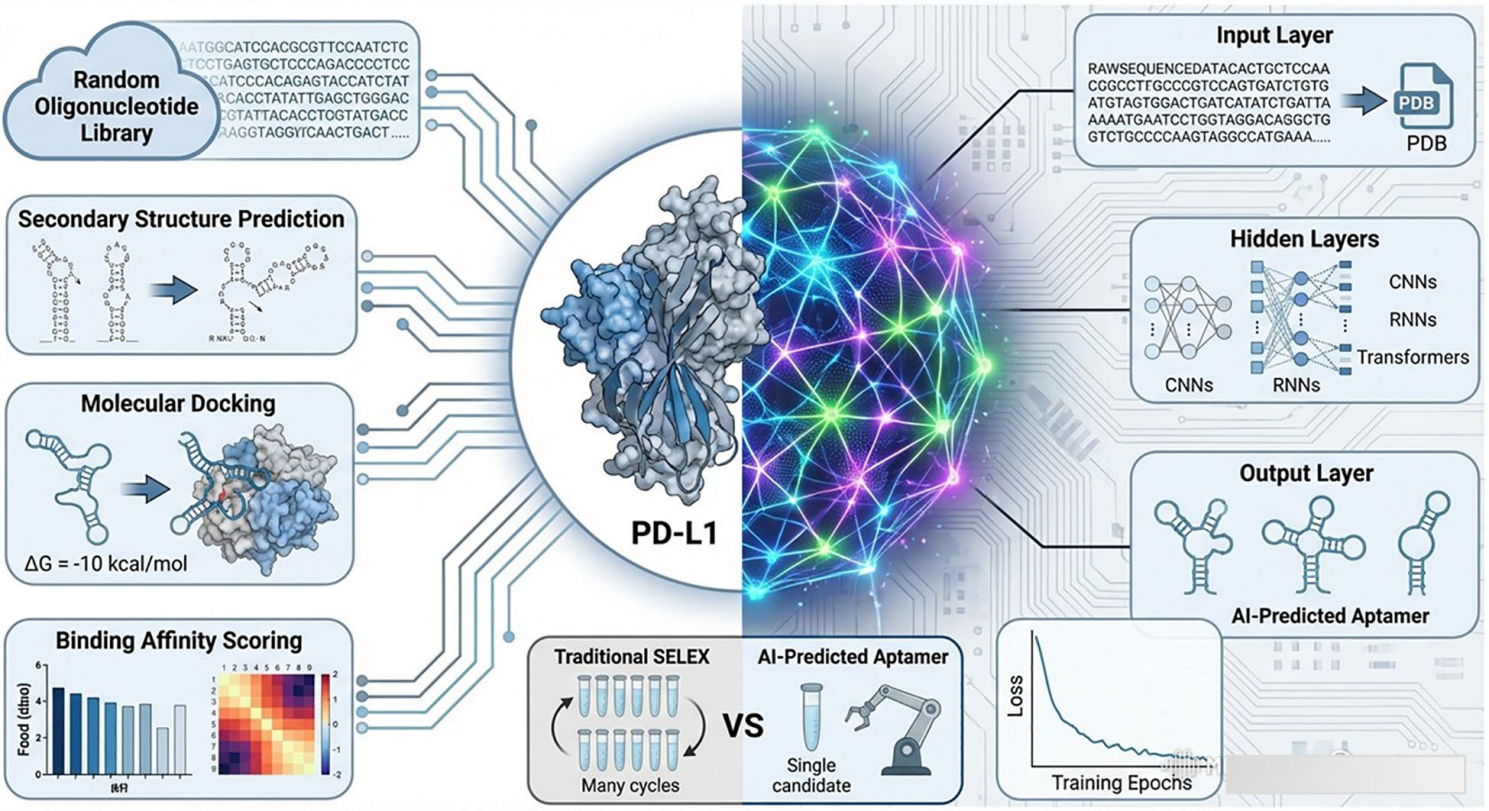
Computational approaches for in-silico and AI aptamer design.

In silico aptamer design relies on computational modeling to predict structures, evaluate binding potential, and prioritize candidates for synthesis and validation, as illustrated in **Table 3**. Secondary structure prediction is a critical first step, as base-pairing patterns, such as hairpins, pseudoknots, and G-quadruplexes, largely dictate the aptamer binding properties. Algorithms, including RNAfold (ViennaRNA), Mfold, RNAstructure, Vfold2D, and CentroidFold, use free energy minimization and comparative approaches to predict secondary structures and identify key motifs, such as stems, loops, and bulges, which form the foundation of tertiary structure folding. Tertiary structure prediction builds on these models to generate three-dimensional aptamer conformations required for detailed binding analyses (39, 40). Tools such as RNAComposer, 3dRNA, Vfold3D, and SimRNA assemble secondary structure elements into 3D models using fragment libraries, motif matching, and coarse-grained force fields. These methods perform well for short sequences (<40nt), although their accuracy declines for longer or more complex aptamers, with Vfold3D often producing the most reliable models for larger constructs. DNA aptamers are commonly modelled by first building RNA structures and then converting them to DNA geometries using molecular visualization tools, such as Chimera or VMD, owing to the lack of specialized DNA structure-predictors. Once the 3D structures are obtained, molecular docking can be used to estimate the binding affinities and interaction modes by simulating aptamer–target complexes. Docking programs such as AutoDock4, AutoDock Vina, ZDOCK, DOCK, and MDockPP explore possible binding configurations and score them using either physics-based or empirical energy functions that account for hydrogen bonding, electrostatics, hydrophobicity and entropy. AutoDock Vina performs particularly well in polar pockets, whereas AutoDock4 performs well in hydrophobic regions. ZDOCK employs fast Fourier transform–based shape complementarity and electrostatic scoring, whereas DOCK and MDockPP rely on force-field terms to identify favorable binding modes. Following docking, molecular dynamics (MD) simulations using engines such as AMBER or GROMACS refine aptamer–target complexes, assess binding stability, and calculate binding free energies using MM-PBSA or MM-GBSA methods (41, 42). These simulations provide dynamic insights that static docking cannot, revealing induced-fit conformational adjustments and persistent interactions over time. In addition to these physics-based methods, machine learning (ML) and deep learning (DL) approaches have been increasingly employed to accelerate aptamer discovery processes. Models such as AptaTrans, which leverage transformer architectures pretrained on protein and nucleotide sequences, can accurately predict aptamer–protein interactions *in silico* (43). Generative algorithms, such as Apta-MCTS, AptaDiff, and RaptGen, can explore vast sequence spaces to propose novel high-affinity candidate aptamers. In contrast, deep neural networks can identify binding patterns even with limited data by leveraging transfer learning (44–46). Together, these computational strategies form a powerful pipeline for aptamer design, enabling the rapid, cost-effective, and rational selection of high-performance sequences before experimental validation (**Figure 7**).

**Table 3:** Bioinformatics tools for aptamer design.

Bioinformatic tool	System requirements	Description and key features
RNAfold (ViennaRNA)	Web server (no installation) or Linux/Windows packages; moderate CPU	Predicts minimum free-energy secondary structures of RNA sequences using thermodynamic models. Provides dot-bracket notation, energy scores, base-pair probabilities, and visualizations. Widely used for initial aptamer structure screening.
Mfold	Web-based; minimal system requirements	Classic secondary structure prediction tool for DNA/RNA. Uses dynamic programming to compute the most stable folding conformations. Provides 2D structure plots and ΔG values.
RNAstructure	Windows/Linux/macOS; GUI and command-line versions available	Predicts RNA/DNA secondary structures with high accuracy; supports pseudoknots, stochastic sampling, and multiple sequence alignment. Useful for identifying conserved motifs in aptamer libraries.
Vfold2D / Vfold3D	Web server or standalone (Linux); moderate CPU for 3D modeling	Vfold2D predicts secondary structures considering loop entropy. Vfold3D constructs RNA 3D models using template-based assembly, performing well for long or complex aptamers.
RNAComposer	Web server; requires stable internet; optional local installation (Linux)	Automates RNA 3D structure modeling from secondary structures. Uses fragment libraries and motif matching to generate full tertiary models. Widely applied for RNA aptamer modeling prior to docking.
3dRNA	Web server or Linux; moderate computational resources	Predicts RNA 3D structures by assembling pre-classified fragments based on secondary structure input. Suitable for generating multiple 3D conformations for docking studies.
AutoDock4 / AutoDock Vina	Windows/Linux/macOS; requires local installation; moderate CPU/GPU for large targets	Performs molecular docking of aptamers to protein or small-molecule targets. AutoDock4 uses physics-based scoring; Vina uses an empirical scoring function for faster computation.
ZDOCK	Linux; requires installation; moderate to high CPU	FFT-based docking tool originally for protein–protein interactions, also applied to aptamer–protein docking. Evaluates shape complementarity and electrostatic interactions.
DOCK	Linux/Unix; command-line; moderate CPU	Early docking software that matches molecular surfaces and evaluates interactions using AMBER-based scoring. Useful for ranking aptamer binding poses.
MDockPP	Linux; requires moderate to high computational resources	Uses FFT-based global docking with a specialized scoring function for protein–ligand interactions, including aptamers. Provides ranked binding modes and interaction energies.
AMBER / GROMACS	High-performance workstation or cluster; Linux recommended; multi-core CPU and GPU supported	Molecular dynamics simulation packages for refining aptamer–target complexes, calculating binding energies (MM-PBSA/MM-GBSA), and analyzing structural stability.
AptaSUITE	Linux/Windows; Java environment required	Analyzes high-throughput SELEX (HT-SELEX) data. Identifies enriched sequences, tracks motif frequency over rounds, and clusters related sequences.
DeepAptamer / AptaTrans	Python (TensorFlow/PyTorch); GPU recommended for training; Linux/Windows	Machine learning frameworks for predicting aptamer–target binding affinities and generating novel aptamer sequences. Use transformer or deep neural architectures.
RaptGen / AptaDiff	Python-based; GPU recommended	Generative deep learning models for de novo aptamer design. Explore large sequence spaces and output high-affinity candidates for further docking and validation.

## 5 Registered clinical trials to assess the efficacy of aptamers in the treatment of cancer

Aptamer-based approaches are emerging as innovative strategies in cancer immunotherapy, leveraging their unique structural versatility, high specificity, and low immunogenicity to modulate immune responses and improve therapeutic outcomes (**Table 4**). Unlike antibodies, aptamers can be chemically synthesized, engineered for multifunctional roles, and modified to enhance their stability and targeting precision. One of the most advanced clinical examples is NOX-A12 (olaptesed pegol), a PEGylated L-RNA Spiegelmer that binds to and neutralizes the chemokine CXCL12 (NCT03168139, OPERA trial, Phase 1/2 open-label study). In patients with microsatellite-stable colorectal and pancreatic cancers (n≈20–30), NOX-A12 was administered as monotherapy, followed by a combination with pembrolizumab. The primary endpoints included safety, pharmacodynamic remodeling of the TME (increased CD8+ T-cell infiltration and reduced immunosuppression via biomarkers), and preliminary efficacy (disease stabilization and objective response rate) (47, 48). The results showed acceptable safety, TME remodeling, and disease stabilization in a subset of patients. Limitations include the small sample size, lack of randomization or control arm, short follow-up, and focus on combination rather than monotherapy checkpoint blockade. No direct PD-1, PD-L1, or CTLA-4 aptamer inhibitors have advanced to late-phase clinical trials. Preclinical candidates targeting these checkpoints show promising antitumor effects in animal models but require further IND-enabling toxicology, pharmacokinetic, and immunogenicity studies before human evaluation. AS1411 (nucleolin-targeting) has been explored in early phase oncology trials, primarily as a delivery scaffold, with mixed safety and efficacy results in small cohorts (49, 50). Collectively, these developments highlight aptamers as powerful tools for reprogramming the tumor microenvironment, enhancing checkpoint blockade, and delivering immunomodulators with high precision, paving the way for their integration into next-generation cancer immunotherapy, although substantial translational gaps remain.

**Table 4:** Clinical trials to assess the efficacy of aptamers in the treatment of cancer immunotherapy.

Aptamer / type	Target / mechanism	Cancer type / application	Clinical status	Immunotherapy role / key outcomes
NOX-A12 (olaptesed pegol)	CXCL12 chemokine trap	Microsatellite-stable colorectal and pancreatic cancer	Phase I/II (OPERA, NCT03168139)	Reprograms tumor microenvironment to enhance T-cell infiltration; combined with PD-1 blockade (pembrolizumab); showed disease stabilization in subsets
PD-1 / PD-L1 Aptamers	Immune checkpoint blockade	Solid tumors (preclinical)	Preclinical	Mimic antibody checkpoint inhibition; activate T cells and promote tumor regression in models
Bispecific Aptamers	Dual tumor antigen and immune receptor targeting	Various solid tumors (preclinical)	Preclinical	Act as T-cell engagers, redirecting immune cells to tumors with high specificity
AS1411 (G-quadruplex DNA)	Nucleolin	Renal cell carcinoma, AML; potential delivery scaffold	Phase I/II (discontinued for RCC due to low response)	Tumor targeting and cellular uptake enhancement; platform for immune cargo delivery
NOX-E36 (emapticap pegol)	CCL2 chemokine trap	Metabolic and inflammatory diseases; preclinical oncology	Clinical (non-oncology); preclinical oncology	Immunomodulation via chemokine neutralization; potential to remodel tumor microenvironment similar to NOX-A12

### 5.1 Safety and toxicity considerations

Nucleic acid therapeutics carry specific risks, including off-target hybridization, rapid renal filtration, potential tubular accumulation (mitigated by PEGylation, size optimization, or nanoparticle encapsulation), and innate immune activation (e.g., TLR9 stimulation by unmethylated CpG motifs leading to cytokine release or complement activation). Preclinical studies of PD-1/PD-L1 and CTLA-4 aptamers have generally reported minimal immunogenicity, low acute toxicity, and no severe organ damage at therapeutic doses. Early clinical experience with pegaptanib and NOX-A12 indicated acceptable tolerability profiles (primarily mild injection-site reactions, fatigue, and transient laboratory abnormalities). However, long-term safety data remain limited, and dedicated monitoring of delayed renal, hepatic, and immune-related effects is essential in future trials.

## 6 Nanoparticle platforms used for aptamer conjugation

**Table 5** depicts the major nanotechnology platforms commonly conjugated with aptamers for targeted therapy and diagnostics. The central structure represents a functionalized nanoparticle bound to an aptamer strand, with surrounding graphical elements highlighting the main nanoparticle categories (51).

**Table 5:** Nanoparticle types used for aptamer conjugation to target cancer immunotherapy.

Nanoparticle platform	Structure & composition	Aptamer conjugation method	Aptamer role / examples	Immunotherapy applications	Advantages	Ref
Liposomes	Phospholipid bilayers enclosing an aqueous core (hydrophilic core + hydrophobic bilayer)	Lipid–PEG spacers with maleimide–thiol or click chemistry	Surface-conjugated aptamers guide liposomes to immune cells or tumor receptors; e.g., PD-L1 aptamer-liposomes for checkpoint blockade; DEC-205 aptamers for dendritic cell targeting	Cytokine delivery, immune checkpoint inhibition, dendritic cell targeting	High drug-loading capacity; clinically established platform; flexible surface chemistry	(51)
Polymeric Nanoparticles	Biodegradable polymers such as PLGA or PEG–PLA forming solid matrices	EDC/NHS covalent coupling or DNA hybridization	Aptamers act as targeting ligands for tumor or immune cells; e.g., AS1411–PLGA NPs for nucleolin targeting; aptamer–siRNA hybrid systems for immune modulation	Sustained delivery of siRNA, mRNA, or immunomodulators to tumors or immune cells	Excellent stability; tunable release kinetics; regulatory familiarity	(52)
Gold Nanoparticles (AuNPs)	Metallic nanospheres exhibiting surface plasmon resonance	Dense attachment via thiol–gold bonds	Aptamers decorate AuNPs for selective tumor targeting and immune checkpoint blockade; e.g., PD-L1 aptamer–AuNPs for localized immunotherapy; aptamer-AuNPs for photothermal therapy	Targeted photothermal ablation inducing immunogenic cell death; local checkpoint blockade; theranostic applications	Simple conjugation; multifunctional (therapy + imaging); strong optical properties	(53)
Silica Nanoparticles	Mesoporous structure with tunable pore sizes and high surface area	EDC/NHS or click chemistry on aminated surfaces with PEG spacers	Aptamers enable selective delivery of immune agonists or antigens; e.g., STING agonist-loaded silica NPs conjugated with tumor-targeting aptamers	Targeted delivery of immune agonists, adjuvants, or antigens; vaccine-like immunotherapy	High loading capacity; rigid structure; multifunctionality	(54, 55)
Solid Lipid Nanoparticles (SLNs) / Nanostructured Lipid Carriers (NLCs)	Lipid-based nanocarriers with solid or mixed solid–liquid cores stabilized by surfactants	Carbodiimide or maleimide coupling; lipid–PEG–aptamer linkers	Aptamers improve targeting of immune modulators to tumor or antigen-presenting cells; e.g., aptamer-NLCs for hydrophobic immune checkpoint inhibitors or adjuvant delivery	Targeted delivery of hydrophobic modulators, small-molecule ICIs, or adjuvants; dendritic cell targeting to boost antigen presentation	High stability; scalable production; enhanced tumor penetration	(56)

### 6.1 Liposomes

Liposomes are spherical vesicles composed of phospholipid bilayers that enclose an aqueous core that can carry hydrophilic (in the core) and hydrophobic (in the bilayer) molecules, respectively. Their surfaces can be modified using lipid–PEG chains with terminal functional groups, allowing aptamer conjugation via maleimide–thiol or click chemistry. PEG spacers help project aptamers outward, reducing steric hindrance and improving the receptor recognition. Free (unconjugated) aptamers exhibit a very short plasma half-life (1h) due to rapid renal clearance and limited tumor retention. In contrast, aptamer-decorated liposomes, polymeric nanoparticles, and gold/silica systems extend circulation time to hours–days, improve tumor accumulation via the enhanced permeability and retention (EPR) effect, enhance penetration in dense stroma, and reduce systemic exposure and off-target effects. Aptamer-decorated liposomes are widely used in cancer immunotherapy for targeted cytokine delivery, immune checkpoint blockade, and dendritic cell targeting. They offer high drug-loading capacity, clinical translation potential, and flexible chemistry, although stability and leakage during storage remain challenging (53).

### 6.2 Polymeric nanoparticles

Polymeric nanoparticles are solid carriers made from biodegradable polymers, such as PLGA or PEG–PLA, offering tunable degradation rates and controlled release profiles. Aptamers can be covalently attached through EDC/NHS chemistry or hybridized with complementary DNA strands anchored to the particle surface (54). These systems are particularly suitable for siRNA, mRNA, or immunomodulator delivery to tumors or immune cells, enabling sustained release over days to weeks after administration. Their advantages include excellent stability, precise control over release kinetics, and regulatory familiarity, whereas their potential limitations include manufacturing reproducibility and degradation-induced acidity, which affect sensitive cargos (55).

### 6.3 Gold nanoparticles

Gold nanoparticles (AuNPs) are metallic nanospheres with unique optical properties owing to localized surface plasmon resonance, which enables their use in photothermal therapy applications. Aptamers conjugate easily and stably to AuNP surfaces through thiol–gold bonds, allowing a dense multivalent display without complicated chemistry (56, 57). In immunotherapy, AuNP–aptamer systems are employed for targeted photothermal ablation, which induces immunogenic cell death and localized checkpoint blockade, or as combination carriers that simultaneously deliver drugs and enable imaging or thermal activation. Their simple conjugation, imaging capabilities, and multifunctionality are major advantages, although their non-biodegradability requires careful consideration for clinical translation (58, 59).

### 6.4 Silica nanoparticles

Mesoporous silica nanoparticles possess a rigid structure with tenable pore sizes and a very high surface area, making them ideal for loading multiple drugs, immune agonists and antigens. Aptamers are typically conjugated to amine-modified silica surfaces using EDC/NHS or click chemistry, often with PEG linkers for improved aptamer presentation. These carriers have been used to deliver STING agonists directly to tumors or antigen-presenting cells and to co-deliver antigens and adjuvants for vaccine-like immunotherapy. Their main strengths are their high loading capacity, structural stability, and multifunctionality, whereas their challenges include their non-biodegradable nature and potential long-term tissue accumulation (60).

### 6.5 Solid lipid nanoparticles and nanostructured lipid carriers

SLNs and NLCs are lipid-based nanocarriers composed of solid or mixed solid–liquid lipid cores stabilized by surfactants, offering a high loading capacity for hydrophobic molecules and good biocompatibility (61, 62). Aptamers are typically conjugated onto the surface using carbodiimide or maleimide chemistry or incorporated using lipid–PEG–aptamer linkers (63). In immunotherapy, aptamer-functionalized NLCs have been employed for the targeted delivery of hydrophobic immune modulators, small-molecule checkpoint inhibitors, or adjuvants, and for directing nanoparticles to dendritic cells to enhance the antigen presentation. Their advantages include excellent stability, scalable production, and enhanced tumor penetration compared to other platforms, although burst release and limited clinical maturity remain challenges (64).

## 7 Aptamers targeted cancer immunotherapy

### 7.1 Aptamer target interactions modulating cancer immunotherapy

Aptamers are known for their remarkable stability across different environments and can be produced at a low cost, making them particularly appealing for use in cancer immunotherapy. Tumor cells frequently avoid detection by the immune system by taking advantage of immune checkpoint receptors, such as TIM-3, LAG-3, CTLA-4, and PD-1. Aptamers can specifically bind to these receptors, blocking the inhibitory signals that dampen T-cell activity, thus restoring the immune system’s ability to identify and destroy cancerous cells. Studies have demonstrated that aptamers targeting PD-1 and PD-L1 can effectively block immune checkpoint pathways, leading to increased T cell activation and enhanced antitumor activity. An anti-CTLA-4 aptamer impedes the suppression of co-stimulatory signals, thereby facilitating T cell proliferation and activation (65). Li et al. reported that aptamers contribute to the maintenance of T-cell stemness and mitigate exhaustion by inhibiting checkpoint signaling, as evidenced by the downregulation of exhaustion-associated gene expression. Furthermore, by mimicking co-stimulatory signals, such as CD28, aptamers promote T-cell activation and the formation of long-term memory (66). Aptamer-based therapies can also be conjugated with nanoparticles or chemotherapeutic agents to enable targeted delivery while minimizing systemic toxicity, as demonstrated by Camorani et al. in triple-negative breast cancer, where aptamer-assisted checkpoint blockade enhanced the therapeutic response and reduced treatment resistance (67). Compared to antibodies, aptamers possess superior tumor penetration capabilities (**Table 6**) and can bind to intracellular targets with high affinity and specificity. Their synthetic nature enables rapid production and structural customization for various cancer types, supporting the development of personalized and cost-effective treatment strategies (68). Aptamers often display nanomolar to picomolar binding affinities owing to their unique three-dimensional architectures that facilitate strong interactions with target molecules via hydrogen bonding, van der Waals forces, and electrostatic interactions (41, 42). High-affinity aptamers targeting PD-1, PD-L1, NKG2A, and CTLA-4 have been reported, with dissociation constants (Kd) measured in nanomolar concentrations, which is a critical parameter for evaluating their therapeutic effectiveness. Heatmap-based affinity analysis aids in identifying aptamers with strong potential for modulating immune checkpoint activity. Binding affinity is influenced by aptamer nucleotide sequence and structural features, including stem-loop formation, which enhances binding stability (69). However, the tumor microenvironment, characterized by hypoxia, low pH, a dense extracellular matrix, and restricted receptor accessibility, may hinder aptamer binding efficiency. The selective targeting of overexpressed receptors or proteins, such as EGFR, nucleolin, and PD-L1, enables precise drug delivery and imaging (70). Chemical modifications, such as the incorporation of 2′-O-methyl or 2′-fluoro residues, recover aptamer stability and affinity under physiological conditions, protecting them from nuclease degradation (71). Structural variations, including hairpin loops, pseudoknots, inner loops, and G-quadruplexes, directly influence the binding performance and target specificity. Binding affinity is commonly assessed using methodologies such as electrophoretic mobility shift assay, isothermal titration calorimetry, and surface plasmon resonance spectroscopy. For instance, an aptamer targeting VEGF demonstrated a Kd of approximately 50 pM using SPR analysis (72). High binding affinity correlates with superior therapeutic efficacy, as observed in thrombin-binding aptamers that exhibit nanomolar Kd values (73). Multi-checkpoint targeting by high-affinity aptamers may more effectively regulate immune responses and overcome tumor-induced immunosuppression than monoclonal antibodies. The interactions of aptamers with immune cells, such as T cells, B cells, NK cells, and macrophages, can be classified as high, moderate, or low, depending on the degree of immune activation or modulation. For example, some aptamers strongly stimulate T cells with moderate macrophage interaction, enhancing cytotoxic responses, whereas others exhibit high modulation of B cells and macrophages, influencing humoral immunity and tumor-associated inflammation. These interaction profiles, visualized using heatmaps, provide insights into aptamer-driven immune modulation and support their potential therapeutic application. Several aptamers have been specifically engineered for cancer immunotherapy to target molecules involved in immune evasion, demonstrating functions such as checkpoint inhibition and T cell activation (**Table 7**).

**Table 6:** Comparison of aptamers and monoclonal antibodies in cancer immunotherapy.

Property / Feature	Aptamers	Monoclonal antibodies	Implications
Molecular size	6–30 kDa	~150 kDa	Aptamers penetrate dense tumors better
Production method	Chemical synthesis	Mammalian cell culture	Aptamers faster, lower cost, no batch variation
Cost	Lower (scalable synthesis)	High (complex bioprocessing)	Aptamers potentially more cost-effective
Immunogenicity	Low	Moderate	Aptamers generally better tolerated
Tissue/tumor penetration	small size	Limited	Advantage in solid tumors with poor vascularity
Chemical modifiability	High	Limited	Aptamers tunable for stability and targeting
Shelf-life / stability	Room temperature possible	Requires a cold chain	Aptamers easier logistics
Manufacturing scalability	High (solid-phase synthesis)	Moderate (but expensive)	Aptamers potentially easier to scale
Clinical maturity (checkpoint blockade)	Preclinical / early-phase (NOX-A12, AS1411)	Mature (pembrolizumab, nivolumab, ipilimumab)	Antibodies currently dominate; aptamers emerging

**Table 7:** Aptamers targeting immune checkpoints and tumor-associated molecules.

Targets	Target cells	Aptamers	Functions	Ref
PD-L1	Tumor cells	AptPD-L1	PD-L1/PD-1 interaction inhibition	(74)
PD-1	T cells	PD-1 Apt1	PD-L1/PD-1 inhibition	(75)
CTLA-4	T cells	CTLA4 Apt	Regulates T-cell activation by competing with CD28 for B7	(76)
CTLA-4 / NKG2A dual receptor	Tumor / NK cells	AXA2T-B2-13	Enhances CD8^+^ T-cell & NK-cell effector functions	(76)
Nucleolin	Tumor cells	AS1411	Inhibits cancer cell proliferation	(77)
PTK7	Tumor cells	Sgc8	Inhibits EGFR signaling	(78)
TIM-3	T cells	S3.1	Blocks TIM-3/galectin-9 interaction	(79)
LAG-3	T cells	SL15	Blocks LAG-3/MHC II interaction	(80)
SDF-1	Tumor cells	NOX-A12 (Spiegelmer)	Binds & neutralizes SDF-1; blocks CXCR4/CXCR7 pathways	(81)
CXCL12	Tumor cells	CXCL12 Apt	Inhibits CXCL12-mediated chemotaxis & metastasis	(82)
VEGF	Vascular endothelial cells	Pegaptanib (Macugen®)	Blocks VEGF-mediated angiogenesis	(83)
TLR9	B cells	CpG7909	Activates immune cells; promotes TNF-α / IL-6 pathway	(84)
TNF-α	Macrophages	SL1026	Blocks IL-6 / IL-6R interaction	(85)
IL-6	Macrophages	SL0026	Blocks IL-6 / IL-6R binding	(86)
IFN-γ	Macrophages	ARC25	Aptamer targeting interferon-γ	(87)

### 7.2 Aptamer-enhancing immune responses mechanism

**Figure 8:**
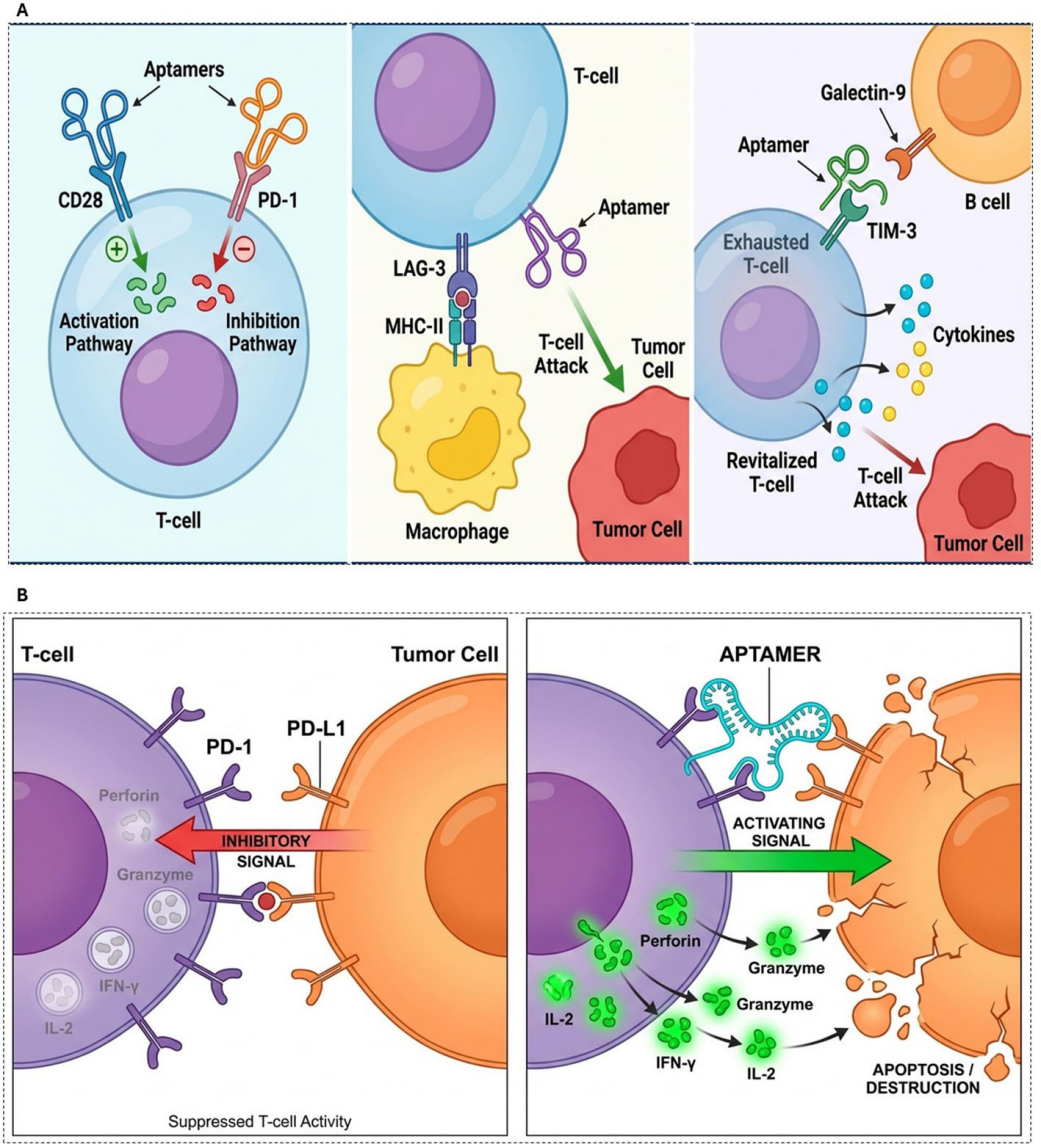
**(A)** General mechanism of immune checkpoint blockade and restoration of T-cell activation (broad co-inhibitory pathway overview. **(B)** Specific molecular mechanism of PD-1/PD-L1 blockade by aptamers (competitive inhibition, prevention of T-cell exhaustion, restoration of cytotoxic signaling).

Cancer immunotherapy is based on the understanding that tumor cells are distinct from normal cells owing to variations in their protein structures and antigen compositions. Immune cells detect tumor-associated antigens as foreign, triggering an immune response aimed at the tumor. Ideally, this response would destroy the tumor cells; however, it often falls short, and tumors develop mechanisms to escape immune detection. These evasion tactics include the secretion of inhibitory ligands that weaken immune function through negative co-stimulation (88, 89). The central goal of cancer immunotherapy is to amplify the immune response against tumors. To improve their effectiveness, strategies have been designed to reduce unwanted immune suppression and enhance signals that bolster antitumor immunity. Traditional therapeutic approaches generally focus on targeting immune receptors, cytokines, and chemokines using antibodies, soluble receptor ligands and recombinant cytokines. Aptamers can interact with the immune system. Initially, research efforts were primarily focused on dampening immune responses to treat autoimmune disorders rather than cancer. However, the use of aptamers in cancer immunotherapy, particularly for enhancing antitumor immunity, has recently attracted considerable interest. Important studies have shown that aptamers influence immune responses via various mechanisms. Certain aptamers enhance innate immunity to boost vaccine effectiveness by improving antigen presentation and T cell activation, often by attaching to pattern recognition receptors on dendritic cells and macrophages. DNA aptamers containing CpG sequences activate Toll-like receptor 9, leading to the production of pro-inflammatory cytokines and immune activation (90, 91). Aptamers can be designed to specifically bind immune molecules or cells, such as cytokines, B cells, and T cells, to regulate immune function. Aptamers that target checkpoint molecules, such as PD-1 and CTLA-4, block the inhibitory signals that prevent T-cell activation, thereby strengthening antitumor immunity (92). Furthermore, aptamers can disrupt immune evasion by interfering with pathogen–immune interactions. Dey et al. developed an aptamer against the HIV-1 gp120 protein to inhibit viral attachment and cellular entry (93). Aptamers have also demonstrated potential in attenuating inflammatory responses by binding to cytokines, chemokines, and their cognate receptors. Aptamers targeting TNF-α and IL-6 can inhibit pro-inflammatory signaling. In contrast, the RNA aptamer pegaptanib suppresses angiogenesis and inflammation by targeting VEGF in patients with age-related macular degeneration (94). In autoimmune disorders, aptamers can suppress abnormal immune activation by targeting autoantibodies or pathogenic immune cells. Wu et al. demonstrated the therapeutic potential of aptamers against IFN-γ for treating rheumatoid arthritis (95). Cancer cells exhibit genetic and epigenetic abnormalities, leading to the production of diverse tumor-associated antigens that are recognized as non-self. However, tumors develop mechanisms to evade immune destruction and modify the TME, promoting their growth and metastasis. Cancer immunotherapy aims to enhance or restore immune function by overcoming these evasion mechanisms. Recent advancements in aptamer-based immunomodulation have shown promise in the treatment of triple-negative breast cancer. Aptamers can recruit immune cells to tumor sites, block immune checkpoint pathways, and enhance the activity of cytotoxic immune cells, making them promising candidates for cancer therapy (96). As illustrated in **Figures 8A**, aptamers bind to targets, such as tumor antigens or immune checkpoints, blocking inhibitory signals and restoring T cell function to enhance antitumor immunity. These results highlight the potential of aptamers in cancer treatment by modulating the immune system. Future research should focus on improving aptamers, combining them with other treatments, and testing their effectiveness in living organisms to make them more useful in medical settings.

### 7.3 Aptamer drug delivery-directed immune regulation

Research on aptamers has intensified owing to their potential to directly influence immune cell activity, target antigens specific to tumors, and transport immune-regulating compounds to cancerous regions. Antigens such as EGFR and HER2 are mainly found on cancer cells, and aptamers can be meticulously designed to identify and attach to these molecules. Upon attachment, aptamers facilitate the targeted delivery of immune-modulating agents, such as IL-2, anti-CTLA-4, anti-PD-L1, anti-PD-1, and IFN-α, directly to tumor sites. This approach supports the localized administration of cytokines, chemotherapeutic agents, or immune checkpoint inhibitors, thereby enhancing therapeutic efficacy and minimizing systemic toxicity and off-target effects (97, 98). Beyond targeted delivery, aptamers can directly alter immune cell behavior by interacting with receptors or signaling molecules on immune cells, such as T lymphocytes, to enhance antitumor immune responses. Specifically, aptamers can be rationally designed to activate stimulatory signaling pathways or inhibit suppressive immune checkpoints, such as PD-1 and CTLA-4, resulting in enhanced T cell activation and cytotoxicity against tumor cells (99). Innovative molecular strategies have further expanded the functionality of aptamers. For instance, a two-uracil (UU) nucleobase linker has been employed to conjugate albumin-binding aptamers to a Dicer substrate double-stranded RNA molecule, forming an aptamer–siRNA chimeric molecule. Following annealing of the siRNA sense strand, these chimeras demonstrated superior siRNA bioavailability and improved *in vivo* stability, contributing to enhanced antitumor immune responses (100). Given their high specificity, low immunogenicity, structural versatility, and ease of chemical modification, aptamers hold remarkable potential for modulating immune cell function and delivering immunotherapeutic agents. They represent innovative alternatives to traditional antibody-based systems and are applicable across various disease contexts, including cancer, autoimmune disorders, and infectious diseases. However, to facilitate successful clinical translation, further research should prioritize the optimization of aptamer stability and delivery, evaluation of combination therapeutic strategies, and validation through preclinical and clinical studies. Addressing these challenges may enable aptamer-based immunotherapies to emerge as transformative modalities in modern medicine.

## 8 Immune checkpoints

Immune checkpoint molecules are critical targets for aptamer-based cancer immunotherapy because they regulate the balance between T-cell activation and exhaustion in the tumor microenvironment. Aptamers have been developed to bind and inhibit PD-1 or PD-L1, effectively blocking the PD-1/PD-L1 interaction and restoring cytotoxic T cell function in a manner analogous to monoclonal antibodies but with superior tissue penetration and reduced immunogenicity (101, 102). Similarly, aptamers targeting CTLA-4 can prevent its inhibitory interaction with CD80/CD86 on antigen-presenting cells, thereby enhancing T cell priming. Emerging aptamers against LAG-3, TIM-3, and TIGIT aim to overcome resistance to single checkpoint blockade by modulating multiple inhibitory pathways (103, 104). Preclinical studies have shown that TIM-3 aptamers restore TCR signaling, LAG-3 aptamers enhance MHC-II interactions, and TIGIT aptamers improve T-cell and NK-cell function by disrupting CD155/CD112 binding. These emerging aptamers may offer cost-effective, low-immunogenicity alternatives to antibodies, although most remain in the early preclinical stages. To facilitate a direct comparison between these two major classes of checkpoint-targeting agents, **Table 6** summarizes the key advantages and limitations of aptamers and monoclonal antibodies in cancer immunotherapy. These aptamers can be used as free inhibitors, tethered to nanoparticles for sustained local release, or incorporated into bispecific constructs to simultaneously target checkpoints and tumor antigens. Their small size enables access to immune-privileged niches, such as poorly perfused tumor cores, offering advantages over bulky antibodies in checkpoint blockade (105, 106).

### 8.1 Aptamer development targeting the PD-1/PD-L1 pathway

The PD-1/PD-L1 axis is a central regulator of immune homeostasis and tumor immune escape, and thus, represents a pivotal target in cancer immunotherapy. Aptamers targeting this pathway provide an innovative therapeutic strategy owing to their high specificity, structural versatility, and tunability. The biological significance of the PD-1/PD-L1 axis and recent advancements in aptamer-based targeting approaches have been well documented (107). **Figure 8B** illustrates how aptamers can effectively inhibit the PD-1/PD-L1 interaction, thereby restoring T cell activity and enhancing cancer cell elimination. High-affinity nucleic acid ligands, such as aptamers, offer advantages over antibodies, including deeper tissue penetration, lower immunogenicity, and ease of chemical modification. While aptamers show strong potential to boost antitumor immunity, challenges remain in optimizing their design and overcoming tumor-associated resistance mechanisms.

#### 8.1.1 PD-1/PD-L1 immune checkpoint

The PD-1 receptor and its ligand PD-L1 are crucial for maintaining immune tolerance by controlling T cell proliferation, cytokine production, and cytotoxic activity. PD-1 is found on activated T cells, whereas PD-L1 is expressed on various immune and tumor cells (108, 109). This interaction is vital for preventing autoimmunity under normal physiological conditions (107). However, tumor cells can manipulate this pathway by overexpressing PD-L1, resulting in an immunosuppressive tumor microenvironment and evasion of immune detection. Consequently, the PD-1/PD-L1 axis has become a key therapeutic target. Monoclonal antibodies (mAbs) targeting PD-1 or PD-L1 have achieved significant clinical success in reactivating T cell-mediated antitumor immunity (110). PD-1 was initially identified by Tasuku Honjo and his team in 1992 as a gene linked to apoptosis; however, further studies have revealed that its role extends beyond regulating cell death. PD-L1 expression, evaluated using immunohistochemistry, has become a predictive biomarker for PD-1/PD-L1 blockade therapy. However, patient responses to treatment vary widely, underscoring the need for reliable biomarkers and strategies to overcome resistance mechanisms (2). In triple-negative breast cancer, targeting the PD-1/PD-L1 checkpoint has shown significant promise in enhancing the patient outcomes. In 2020, the FDA approved pembrolizumab in combination with nab-paclitaxel as a first-line treatment for metastatic TNBC. However, clinical response rates range from 15% to 60%, reflecting the complexity of the tumor microenvironment and the variability in treatment outcomes (111). Additional challenges include high costs, therapy resistance, and immune-related adverse events, prompting research into alternative treatments, such as aptamer-based therapeutics.

#### 8.1.2 Aptamers target in PD-1/PD-L1

Aptamers present a compelling alternative to the challenges associated with current PD-1/PD-L1 targeting therapies. These short, single-stranded oligonucleotides demonstrate outstanding specificity and affinity and successfully block checkpoint signaling. Owing to their smaller size compared to antibodies, aptamers can penetrate tissues more effectively, even in densely packed tumor microenvironments. Their synthetic nature permits precise chemical modifications that enhance structural stability, bioavailability, and resistance to nuclease degradation, while reducing immunogenicity (112). Recent advances have focused on refining aptamer binding characteristics, including structure-based engineering, to reinforce interaction strength. One strategic approach involves the design of aptamers that mimic PD-1, competitively inhibit PD-L1 binding, and restore T cell activity. Integration with nanoparticle-based systems has further improved the therapeutic delivery. Camorani et al. demonstrated significant tumor suppression in a TNBC mouse model using PD-L1-targeting aptamers conjugated with immunostimulatory agents (86). Early phase clinical studies are currently assessing the safety and therapeutic potentials of these agents. Although still in development compared to antibody-based agents, preliminary results indicate favorable efficacy with reduced adverse effects, supporting the advancement of personalized aptamer-based immunotherapy (86).

#### 8.1.3 PD-1/PD-L1 aptamers with monoclonal antibodies compressions

##### 8.1.3.1 Efficacy

Both monoclonal antibodies and aptamers effectively inhibit the PD-1/PD-L1 interaction, restoring T cell activity and enhancing antitumor immune responses. Clinically approved mAbs, such as pembrolizumab and nivolumab, have achieved durable outcomes in malignancies, including melanoma and non–small cell lung cancer (113). However, aptamers offer several key advantages. Their small molecular size facilitates enhanced penetration into solid tumors, potentially improving therapeutic performance in poorly accessible environments. Additionally, Aptamers can be chemically modified to enhance their binding affinity and stability in challenging physiological environments. Preclinical research has demonstrated that aptamers targeting PD-L1 can inhibit tumor growth as effectively as, or even better than, monoclonal antibodies (mAbs), while also showing reduced immunogenicity (**Table 6**). The FDA-approved aptamer drug pegaptanib (Macugen) exemplifies the clinical promise of nucleic acid-based therapies, having successfully treated age-related macular degeneration (114).

##### 8.1.3.2 Safety profiles

While monoclonal antibodies are effective, they are known to cause significant immune-related adverse events (irAEs), such as pneumonitis, colitis, and endocrinopathies, due to overactive immune responses. Furthermore, they can lead to Cytokine Release Syndrome and hypersensitivity reactions, including symptoms such as fever, chills, and low blood pressure, which present clinical challenges (115, 116). Furthermore, variability in toxicity has been reported across checkpoint inhibitors; for example, ipilimumab (anti-CTLA-4) and pembrolizumab/nivolumab (anti-PD-1/PD-L1) can cause organ-specific inflammatory responses. The development of anti-drug antibodies may further compromise efficacy and precipitate allergic reactions (117). In contrast, aptamers demonstrate lower immunogenicity owing to their non-proteinaceous nature and are typically rapidly cleared from circulation when unbound, limiting systemic toxicity (118).

##### 8.1.3.3 Cost Implications

The production of monoclonal antibodies involves complex bioprocessing using living cell systems and rigorous quality control, contributing to high manufacturing costs and limiting accessibility, particularly in resource-limited settings. However, aptamers are chemically synthesized, supporting scalable and cost-efficient production (119). In addition, their shorter developmental timelines offer economic advantages. Aptamer-based therapeutics are a more accessible alternative, potentially enhancing global equity in cancer care while reducing healthcare expenditures.

### 8.2 Cytotoxic T-lymphocyte-associated antigen 4 aptamers

Cytotoxic T-lymphocyte-associated antigen-4 is one of the earliest discovered immune checkpoint molecules, playing a crucial role in maintaining immune tolerance. It competes with the co-stimulatory receptor CD28 to bind to B7 ligands on antigen-presenting cells, thereby inhibiting T cell activation and proliferation. While this mechanism is essential for preventing autoimmune diseases, tumor cells exploit it to evade immune detection and destruction. Immune checkpoint inhibitors targeting CTLA-4, such as ipilimumab, have shown significant therapeutic benefits, particularly in the treatment of melanoma. Aptamers targeting CTLA-4 offer a novel therapeutic approach by specifically disrupting the CTLA-4/B7 ligand interaction, thereby reactivating T cell function. Current research focuses on improving the stability, targeting precision, and bioavailability of CTLA-4 aptamers. Bertrand et al. advanced the development of RNA-based aptamers that penetrate tumors more effectively than antibodies. Moreover, CTLA-4 aptamers exhibit lower immunogenicity, which is advantageous for patients requiring repeated or prolonged treatment, a common limitation of monoclonal antibody therapy. These advantages position CTLA-4 aptamers as promising candidates for future immunotherapeutic approaches.

### 8.3 TIM-3 and LAG-3 aptamers

T-cell immunoglobulin and mucin-domain containing-3 (TIM-3) and lymphocyte activation gene 3 (LAG-3) are newly recognized immune checkpoint receptors that contribute to tumor-induced immune system suppression (37). TIM-3 interacts with galectin-9, leading to the inhibition of T cell receptor signaling and resulting in T cell exhaustion, a common feature of chronic infections and cancer. LAG-3 binds to major histocompatibility complex class II (MHC II) molecules, thereby weakening antigen-specific T-cell responses. Notably, tumor-infiltrating lymphocytes often express TIM-3, LAG-3, and PD-1. Elevated levels of these checkpoints have been observed in T cells from mice that developed resistance to anti-PD-1 therapy in a lung adenocarcinoma model (120). The development of aptamers targeting TIM-3 and LAG-3 has opened new possibilities for immunotherapy. TIM-3 aptamers are designed to disrupt the TIM-3/galectin-9 interaction, thereby reversing T cell exhaustion and enhancing antitumor immune responses. Garcia Melian et al. presented promising preclinical evidence supporting the efficacy of TIM-3 aptamers, particularly when used in conjunction with PD-1 inhibitors. LAG-3 aptamers have also garnered attention because of their potential to modulate immune suppression within the tumor microenvironment. Compared to monoclonal antibodies, LAG-3-targeting aptamers can be chemically optimized to improve tissue penetration and extend therapeutic activity. Ayass et al. highlighted the potential of LAG-3 aptamers to work synergistically with other checkpoint inhibitors to enhance overall treatment outcomes. In cancer immunotherapy, aptamers targeting CTLA-4, TIM-3, and LAG-3 may offer superior alternatives to monoclonal antibodies because they effectively block T-cell suppressive signals and restore antitumor immunity. Despite promising preclinical results, challenges remain in terms of pharmacokinetics, binding affinity, and *in vivo* stability. Future research should focus on translating these experimental advances into clinical applications to fully harness the therapeutic potential of aptamer-based checkpoint blockade.

### 8.4 Other immune checkpoints and associated aptamers

#### 8.4.1 Co-stimulatory molecules

Targeting co-stimulatory receptors with aptamers is a powerful method for boosting antitumor immunity by enhancing T cell proliferation, survival, and effector function. Agonistic aptamers against receptors, such as 4-1BB (CD137) and OX40 (CD134), have been shown to mimic natural ligand binding and induce potent T cell co-stimulation, leading to enhanced cytotoxicity against tumor cells (121, 122). Aptamer dimers or multivalent formats are often employed to achieve receptor clustering, which is essential for co-stimulatory signaling in T-cells. CD28 and CD40 are additional critical targets; aptamers engaging CD28 can boost T-cell activation synergistically with TCR stimulation, while CD40-targeting aptamers can activate dendritic cells and B cells to improve antigen presentation and priming of adaptive immunity. Unlike systemic agonist antibodies, which are associated with toxicity, aptamer-based agonists can be localized to the tumor site or co-delivered with antigens on nanoparticles to minimize systemic immune activation and improve the therapeutic index.

#### 8.4.2 Cytokines and chemokines

Cytokines and chemokines are pivotal regulators of immune cell trafficking, activation, and differentiation, making them key targets for aptamer-mediated modulation of immune responses. Aptamers can bind to cytokines/chemokines (acting as antagonists to block immunosuppressive signaling) or target cytokine receptors to enhance the immune response. For example, aptamers directed against CXCL12 or its receptor CXCR4 can inhibit chemokine-mediated recruitment of immunosuppressive cells, thereby improving T cell infiltration into tumors. Aptamers targeting IL-2 receptors have been explored to selectively stimulate effector T cells over regulatory T cells, thereby improving the therapeutic window of IL-2-based immunotherapy (123, 124). Additionally, aptamers can serve as targeting ligands on nanoparticles carrying cytokine IL-12 to tumor sites, achieving localized immune activation while avoiding systemic cytokine release. This dual ability to block or enhance signaling makes cytokine/chemokine aptamers versatile tools for reshaping immune responses.

#### 8.4.3 Tumor-associated antigens

Tumor-associated antigens provide highly specific molecular signatures that can be targeted by aptamers for selective drug and immunomodulator delivery to tumor cells. Well-characterized aptamers against prostate-specific membrane antigen, epithelial cell adhesion molecule (EpCAM), HER2, and MUC1 have been developed to direct immune effectors or therapeutic nanoparticles specifically to tumor cells. Nucleolin, a cell-surface protein overexpressed in many cancers, is another frequently exploited target for aptamer binding (125). By engaging TAAs, aptamer constructs can deliver checkpoint inhibitors, co-stimulatory agonists, cytokines, or siRNAs directly to tumor cells, enhancing local immune modulation and minimizing the off-target effects. TAA-targeted aptamers can also be engineered as bispecific adaptors, simultaneously binding a TAA on tumor cells and an immune receptor (CD8 or CD3) on T cells, thereby physically bridging immune cells to tumors and facilitating the formation of immune synapses. This strategy is reminiscent of bispecific antibodies but offers greater design flexibility.

#### 8.4.4 Immune cell surface markers

Aptamers targeting immune cell surface markers enable precise modulation of immune subsets to fine-tune antitumor responses. CD8- and CD4-specific aptamers can be used to deliver activating signals, cytokines, or adjuvants directly to cytotoxic or helper T cells, respectively, without affecting the bystander cells. Aptamers against DEC-205, CD40, and CD11c have been used to direct antigens or immune stimulants to dendritic cells, promoting efficient antigen uptake, processing, and cross-presentation to T cells. Such targeted delivery is particularly useful for cancer vaccination strategies, allowing antigen–aptamer–nanoparticle constructs to prime strong adaptive immune responses with lower doses and reduced systemic toxicity compared to conventional vaccines (126). By selectively engaging immune cell markers, aptamers serve as precision delivery vehicles that can activate, suppress, or reprogram specific immune subsets within the tumor microenvironment, thereby complementing the checkpoint blockade and co-stimulatory approaches. **Table 7** summarizes the current advances in the application of aptamers for cancer immunotherapy, emphasizing their roles as molecular-targeting agents, immune modulators, and delivery vehicles. Aptamers offer several advantages over monoclonal antibodies, including a smaller size, reduced immunogenicity, high binding specificity, ease of chemical modification, and improved tumor penetration. These features make aptamers highly promising candidates for next-generation immunotherapeutic approaches.

#### 8.4.5 Clinical applications

Monoclonal antibodies (mAbs) are extensively used to treat infectious diseases, autoimmune conditions, and cancer. Notable examples include trastuzumab (Herceptin) for HER2-positive breast cancer and adalimumab (Humira) for rheumatoid-articular arthritis. Although antibody therapies are well-established in clinical settings, aptamers are gaining attention as highly promising agents for both diagnosis and treatment. The FDA-approved aptamer pegaptanib (Macugen) is currently used to manage age-related macular degeneration (AMD), demonstrating the clinical viability of nucleic acid-based therapeutics (127). Aptamers are actively being explored for precision drug delivery and immune modulation in cancer treatment. Their small molecular size, low immunogenicity, rapid tissue penetration, and ease of chemical modification support their potential as complementary or alternative agents to monoclonal antibodies. **Table 8** provides a comparative overview of PD-1/PD-L1 aptamers and mAbs. While antibodies possess proven clinical efficacy and established safety profiles, aptamers offer advantages such as scalable synthesis, structural flexibility, and potentially lower production costs. However, aptamers require further optimization to improve their stability, pharmacokinetics, and *in vivo* performance. Despite these challenges, their high binding affinity and comparable inhibitory effects observed in preclinical models make them promising candidates for cancer immunotherapy.

**Table 8:** Comparison of monoclonal antibodies (mAbs) vs PD-1/PD-L1 aptamers.

Parameter	Monoclonal antibodies (mAbs)	PD-1/PD-L1 aptamers
Therapeutic efficacy	Demonstrate strong and sustained blockade of the PD-1/PD-L1 axis, producing durable anti-tumor responses in cancers such as melanoma and NSCLC.	Achieve comparable PD-1/PD-L1 inhibition with markedly improved penetration into solid tumors.
Safety profile	Frequently associated with immune-related adverse events (irAEs), including colitis and pneumonitis, owing to broad systemic immune activation.	Exhibit minimal irAEs and reduced off-target effects; lower likelihood of systemic immune activation or allergic reactions.
Immunogenicity	High immunogenic potential due to their protein nature, increasing risks of allergic responses and anti-drug antibody formation.	Low immunogenicity because they are synthetic nucleic acid molecules; generally, well tolerated during repeated administration.
Production cost	Production is costly due to mammalian cell-based bioprocessing, purification, and stringent quality control requirements.	Significantly more economical, as chemical synthesis enables scalable, reproducible, and cost-effective manufacturing.
Development timeline	Requires lengthy development cycles involving antibody screening, optimization, and cell-based production workflows.	Rapid development enabled by computational design, SELEX optimization, and high-throughput screening technologies.
Stability and handling	Depend on cold-chain storage and exhibit reduced stability outside controlled environmental conditions.	Highly stable and chemically tunable; capable of withstanding broader environmental conditions and longer storage durations.
Cost to patients	Treatment expenses are extremely high (USD 100,000–150,000 annually), limiting access for many patients.	Substantially lower treatment costs, improving accessibility, particularly in resource-limited settings.
Clinical accessibility	Widely approved and routinely used across multiple cancer types; central to current immunotherapy regimens.	Primarily in preclinical or early clinical evaluation, though showing strong potential for future translation.
Tissue penetration	Large molecular size restricts diffusion into dense tumor tissue and reduces intratumoral penetration.	Small size and structural flexibility confer superior penetration into solid tumor microenvironments.
Environmental impact	High environmental footprint due to large-scale biologics manufacturing, energy usage, and waste generation.	Lower environmental burden, as production relies on purely chemical synthesis without animal-derived systems.

#### 8.4.6 Immune checkpoint aptamer clinical progress

Immune checkpoint aptamers are a rapidly advancing class of therapeutic agents that function similarly to monoclonal antibodies but have notable advantages, including lower immunogenicity, simpler manufacturing, and enhanced tissue penetration. Preclinical and early clinical investigations have shown compelling results, particularly for PD-1 and PD-L1 aptamers. Zhou et al. reported that PD-1-targeting aptamers effectively blocked PD-1/PD-L1 interactions, induced cytokine production, and promoted T cell proliferation in murine tumor models. Similarly, PD-L1 aptamers conjugated with nanoparticles have demonstrated enhanced tumor suppression through targeted delivery in preclinical studies (137). Beyond the PD-1/PD-L1 axis, CTLA-4 aptamers have shown promise as alternatives to anti-CTLA-4 antibodies. Lee et al. demonstrated that CTLA-4 aptamer treatment inhibited regulatory T-cell activity and enhanced antitumor immunity in melanoma models (136). To further strengthen therapeutic outcomes, bispecific aptamers targeting multiple checkpoints (PD-1 and CTLA-4) have been developed, exhibiting synergistic antitumor effects in preclinical studies (89, 138). TIM-3 aptamers have also shown potential to reverse T-cell exhaustion and improve antitumor responses, particularly when used in combination with PD-1/PD-L1 blockade therapy. Garcia Melian et al. reported improved immune activation using TIM-3 aptamers in murine models (139). Additionally, LAG-3 aptamers designed to block the LAG-3/MHC-II interaction demonstrated enhanced T-cell activation and acted synergistically with PD-1 inhibitors in animal models (140). Although still in the early stages of clinical translation, rapid advancements in immune checkpoint aptamer research demonstrate significant potential. Continued optimization of structural stability, delivery strategies, and pharmacokinetics, along with rigorous clinical evaluation, is crucial for their successful implementation. With further development, aptamer-based immune checkpoint inhibitors may offer highly precise, cost-effective, and patient-tailored alternatives to traditional antibody-based therapies for cancer immunotherapy.

#### 8.4.7 Developing targets in tumor immunotherapy

In cancer treatment research, the identification and targeting of new molecules that control the immune system are becoming increasingly important. Scientists are studying new targets, such as TIGIT, VISTA, and B7-H3, to determine how they affect the immune system and cancer growth. The development of aptamers for these targets is novel but promising. TIGIT is a receptor on T and natural killer (NK) cells. It interacts with CD155 and CD112 on other cells, including cancer cells. TIGIT helps regulatory T cells (Tregs) and stops T cell activation, allowing cancer cells to evade the immune system (141). Studies have shown that blocking TIGIT and using radiotherapy can improve the immune response against tumors. This occurs by activating certain pathways and increasing protein release. After radiation, the levels of TIGIT and CD155 increase in T cells and dendritic cells. Aptamers that target TIGIT can block its interaction with CD155/CD112, thereby enhancing the function of T cells and NK cells. These aptamers may be cheaper and cause fewer immune reactions than antibody treatments. VISTA is another checkpoint for T and myeloid cells. It prevents T cell activation, helping to maintain immune balance. Aptamers targeting VISTA may block its signals, allowing T cells to work again and fight cancer better. Similarly, B7-H3 is a co-inhibitory molecule expressed on both cancer and immune cells that contributes to immune evasion by suppressing T-cell activation. Aptamers directed against B7-H3 can potentially block this interaction, reverse tumor-induced immune suppression, and promote antitumor responses (142). Current efforts are focused on designing aptamers that effectively bind to and inhibit these receptors to counter tumor resistance mechanisms. Bi et al. highlighted the scalability and cost-effectiveness of aptamer synthesis as key benefits in the development of therapeutics for these new checkpoints. Furthermore, the potential to link aptamers with imaging agents or therapeutic payloads offers a promising strategy for personalized medicine, facilitating both targeted drug delivery and real-time tracking of therapeutic outcomes in personalized medicine. While most immunotherapies aimed at TIGIT, VISTA, and B7-H3 are still in preclinical or early clinical trial stages, preliminary evidence indicates their therapeutic potential. However, the available data on aptamers targeting these emerging checkpoints are limited. Continued investigation, structural refinement, and rigorous clinical development are critical for fully harnessing the potential of aptamer-based strategies for next-generation cancer immunotherapy (143, 144).

## 9 Challenges and limitations in aptamer development

Aptamers have shown considerable promise in tumor immunotherapy due to their exceptional specificity, adaptability, and low immunogenicity, especially when targeting immune checkpoint molecules like TIM-3, LAG-3, PD-1/PD-L1, and CTLA-4. Nonetheless, their clinical application faces several obstacles. Addressing these limitations through advances in aptamer engineering and delivery technologies is essential for fully harnessing their therapeutic capabilities (128, 129).

### 9.1 *In vivo* stability and pharmacokinetics

A major obstacle is the *in vivo* instability. Aptamers are susceptible tonuclease-mediated degradation, resulting in a reduced half-life and diminished therapeutic efficacy. The incorporation of chemical modifications, such as 2′-fluoro or 2′-O-methyl substitutions, improves nuclease resistance but may adversely affect binding affinity or target specificity. Free (unconjugated) aptamers exhibit a very short plasma half-life (<1 h) owing to rapid renal clearance and limited tumor retention. In contrast, PEGylation, nanoparticle conjugation (e.g., liposomes, polymeric nanoparticles, gold/silica systems), or size-increasing modifications extend circulation time to hours–days, improve tumor accumulation via the enhanced permeability and retention (EPR) effect, enhance penetration in dense stroma, and reduce systemic exposure and off-target effects, as summarized in **Table 9**. Despite these advances, the degradation kinetics and long-term biodistribution profiles of many nanoparticle platforms (quantum dots, MXenes, upconversion nanoparticles) remain incompletely understood, necessitating further pharmacokinetic and toxicological studies (130, 131).

**Table 9:** Modifications over aptamer addressing nuclease degradation and poor bioavailability.

Modification type	Added agents	Rewritten contributions	Ref
Chemical modifications	2′-O-Methyl (2′-OMe), 2′-Fluoro (2′-F), 2′-Amino (2′-NH_2_)	Enhance resistance to nuclease-mediated degradation while preserving high binding affinity.	(128)
	PEGylation	Improves structural stability and extends the aptamer’s circulation time in vivo.	(129)
Molecule conjugation	Protein (albumin)	Improves pharmacokinetic behavior and reduces rapid clearance.	(130)
	Lipid	Boosts bioavailability and promotes better cell membrane interaction and uptake.	(131)
Nanoparticle encapsulation	AuNPs (gold nanoparticles)	Provides structural protection and enhances intracellular uptake efficiency.	(132)
	Polymeric carriers & liposomes	Shields aptamers from enzymatic degradation and facilitates targeted tissue delivery.	(133)
Spiegelmers	L-enantiomers	Greatly increases nuclease resistance due to their unnatural stereochemistry.	(134)
Optimization sequence	Computational design tools	Produces aptamers with improved folding, higher affinity, and greater overall stability.	(135)
Drug conjugation	Therapeutic molecules (Dox, Dau)	Enhances aptamer durability and enables targeted therapeutic delivery with increased efficacy.	(136)

### 9.2 Safety, toxicity, and off-target effects

Nucleic acid therapeutics carry specific risks including off-target hybridization, rapid renal filtration and potential tubular accumulation (mitigated by PEGylation, size optimization, or nanoparticle encapsulation), and innate immune activation (e.g., TLR9 stimulation by unmethylated CpG motifs leading to cytokine release or complement activation). Preclinical studies of PD-1/PD-L1 and CTLA-4 aptamers generally report minimal immunogenicity, low acute toxicity, and no severe organ damage at therapeutic doses. Early clinical experience with pegaptanib and NOX-A12 indicates acceptable tolerability profiles (primarily mild injection-site reactions, fatigue, transient laboratory abnormalities). However, long-term safety data remain limited, and dedicated monitoring for delayed renal, hepatic, and immune-related effects is essential in future trials. As immune checkpoint molecules are present on both cancerous and normal immune cells, incorrect targeting may result in autoimmune-like effects, necessitating refined specificity. Additionally, the potential for unintended interactions with normal tissues or immune cells highlights the need for rigorous epitope mapping and specificity validation during aptamer development (132, 133).

### 9.3 Regulatory and developmental challenges

Aptamer therapeutics follow the regulatory pathways established for synthetic oligonucleotide drugs (FDA/EMA guidance on INDs for nucleic acid-based products). Key considerations include detailed CMC characterization (sequence integrity, modification uniformity, purity, and scalability), nonclinical pharmacology/toxicology in relevant species, robust immunogenicity assays, and clinical trial designs emphasizing early safety endpoints, pharmacodynamic biomarkers, immune monitoring, and long-term follow-up for delayed adverse events. Challenges include limited clinical precedents beyond pegaptanib and NOX-A12, high costs of large-scale GMP synthesis, batch-to-batch consistency, and extended timelines for regulatory approval owing to the novelty of aptamer-based immunotherapy. Manufacturing reproducibility, especially for chemically modified or nanoparticle-conjugated aptamers, remains technically challenging and may increase costs if inefficiencies persist. Furthermore, the absence of established comparability protocols for post-approval changes and the requirement for sensitive, validated assays to detect anti-aptamer immune responses add complexity to the development pathway (134, 135).

## 10 Future directions and advancements in aptamer engineering

Recent advancements in aptamer engineering have accelerated progress toward overcoming these limitations. Enhanced selection techniques, particularly cell-based systematic evolution of ligands by exponential enrichment (cell-SELEX), have enabled the development of high-affinity aptamers that target tumor-associated molecules. Artificial intelligence (AI) and machine learning (ML) approaches, including deep learning models (AptaNet and SMART-Aptamer), are now used to predict binding affinities, optimize sequences, and accelerate discovery, significantly reducing the reliance on purely empirical methods. Melian et al. emphasized the use of optimized SELEX protocols adapted for immune checkpoint molecules to improve their binding characteristics. Despite significant technical improvements, the experimental identification of aptamers remains laborious and time-consuming. To address this issue, structure-based approaches are increasingly being used in computationally guided aptamer design. Fallah et al. applied a structure-based strategy to engineer high-affinity aptamers, while Sun et al. utilized computational platforms to accelerate aptamer selection and refinement (145, 146).

Artificial intelligence (AI) and computational biology have revolutionized the discovery of aptamers. Machine learning (ML) and deep learning (DL) algorithms are used to predict binding affinities based on large datasets of aptamer–target interaction profiles. Wu et al. successfully employed a deep learning model to identify high-affinity aptamer sequences against cancer biomarkers (147). Emami et al. introduced AptaNet, a deep neural network combining k-mer and inverse complementary k-mer encoding to predict aptamer–protein interaction pairs, demonstrating its application in aptamer sequence optimization (148–152). Similarly, Yang et al. used machine learning to identify novel aptamer–protein interactions by integrating aptamer nucleotide characteristics and protein sequence data with sparse autoencoders. Song et al. developed SMART-Aptamer, an unsupervised machine learning-based multi-dimensional analysis platform that utilizes high-throughput sequencing (HTS) data from SELEX libraries. This system successfully identified high-affinity aptamers against hESCs, EpCAM, and CSV with reduced false discovery rates. These computational tools significantly reduce the dependence on empirical methods, enabling rapid screening, structural optimization, and identification of novel multifunctional aptamers. Personalized medicine represents a major future direction for tumor immunotherapy using aptamers. Owing to their ability to adapt to sequences, aptamers tailored to individual patients can be created to specifically target distinct tumor antigens or immune checkpoint profiles. Aptamer–nanoparticle conjugates and bispecific engagers (targeting tumor antigens and immune receptors simultaneously) are being explored for precise immunomodulation and theranostic applications. This enhances the therapeutic accuracy and minimizes unintended effects. Another promising approach is combination therapy, in which aptamers are used in conjunction with traditional immune checkpoint inhibitors or chemotherapy drugs. Ayass et al. demonstrated improved outcomes with PD-1 aptamer–drug conjugates (ApDCs) in preclinical studies. Additionally, aptamer–nanoparticle conjugates offer the potential to deliver multiple therapeutic agents or genetic materials directly to the tumor microenvironment. Aptamer-based biosensors have been extensively explored for early cancer detection using technologies such as electrochemical, fluorescence, colorimetric, and microfluidic assays. In electrochemical biosensors, aptamers fixed on the electrode surfaces identify cancer biomarkers through signal changes. Quazi et al. developed an electrochemical aptamer biosensor targeting MUC1, achieving a detection limit of 0.0038 pM in breast cancer samples. Fluorescence biosensors utilize aptamers tagged with fluorophores and quantum dots (QDs). Zhang et al. developed aptamer-functionalized Zn^2+^-doped CdTe QDs for imaging of MUC1-overexpressing tumor cells, demonstrating high photostability, reduced toxicity, and precise *in vivo* targeting. In a preclinical model, Mn_3_O_4_@SiO_2_(RB)-PEG-Apt nanoparticles conjugated with AS1411 aptamers targeting nucleolin enabled efficient MRI visualization and biodistribution in HeLa tumor-bearing mice (153–158). Colorimetric biosensors allow for visible detection and are advantageous for point-of-care diagnostics. Geleta et al. demonstrated a colorimetric aptamer-based system for detecting toxic microbial metabolites, which was superior to conventional methods in terms of rapidity, affordability, and ease of interpretation. To improve throughput and specificity, Sheng et al. developed a microfluidic aptamer-based system capable of isolating leukemia cells with 95% capture efficiency and 81% purity, processing 1 mL of blood in 28 min. Furthermore, microheater-assisted temperature-controlled microfluidic platforms using sgc8c aptamers have demonstrated targeted cell isolation and minimal viability loss during release (158–161).

Recently, Achiko et al. introduced a saliva-based SARS-CoV-2 diagnostic platform that combines DNA aptamer biosensing with digital health passport integration, demonstrating rapid and low-cost disease detection. Such innovations highlight the potential of aptamers in theranostic applications, aligning with the goals of precision oncology for minimally invasive, highly targeted therapies. Standardized clinical protocols are essential for ensuring the reproducibility, safety, and reliability of aptamer-based tools. Studies must employ rigorously defined patient selection criteria, monitor experimental conditions, and use robust validation frameworks (87, 162). The key outcome measures included safety (toxicity and adverse reactions), therapeutic efficacy (clinical improvement or biomarker reduction), pharmacokinetics (absorption, distribution, metabolism, and elimination), and diagnostic accuracy (sensitivity, specificity, and predictive value). Additionally, scalability and cost-effectiveness assessments are critical for practical applications in clinical settings (163–165).

## 11 Conclusion

This review provides a thorough analysis of the progress and challenges in the development of aptamers for tumor immunotherapy, with a particular focus on immune checkpoint blockade. By exploring the PD-1/PD-L1 axis and emerging targets such as TIM-3, LAG-3, and CTLA-4, this review highlights the superior specificity, modularity, and reduced immunogenicity of aptamers compared to monoclonal antibodies. Their chemical adaptability enables the creation of multifunctional therapeutic formats, including bispecific aptamers, aptamer–drug conjugates (ApDCs), and aptamer–nanoparticle systems, thereby broadening their therapeutic applicability across various diseases. Nonetheless, significant challenges persist, particularly concerning *in vivo* stability and large-scale manufacturing processes. This review emphasizes the importance of advanced SELEX techniques, structure-based optimization, and AI-driven aptamer design in overcoming these challenges. Furthermore, the integration of aptamers into combination immunotherapy strategies represents a promising avenue for enhancing treatment efficacy and supporting personalized cancer therapies. Overall, aptamers are emerging as potent and adaptable tools for cancer immunotherapy, offering precise and customizable solutions for immune modulation. Their successful clinical translation will depend on continued interdisciplinary collaboration across molecular biology, computational sciences, and clinical research.
